# Nitric oxide-driven Warburg reprogramming at the NOS2-COX2 axis: An integrative engine of cancer hallmarks

**DOI:** 10.1016/j.redox.2026.104245

**Published:** 2026-06-02

**Authors:** Leandro L. Coutinho, Erika M. Palmieri, Lisa A. Ridnour, Robert Y.S. Cheng, William F. Heinz, Stephen K. Anderson, Timothy R. Billiar, Jenny Chang, Stephen J. Lockett, M. Cristina Rangel, Douglas D. Thomas, Daniel W. McVicar, David A. Wink

**Affiliations:** a Cancer Innovation Laboratory, Center for Cancer Research, National Cancer Institute, National Institutes of Health, Frederick, MD, USA; b Optical Microscopy and Analysis Laboratory, Cancer Research Technology Program, Frederick National Laboratory for Cancer Research, Frederick, MD, USA; c Department of Surgery, University of Pittsburgh Medical Center, Pittsburgh, PA, USA; d Houston Methodist Neal Cancer Center, Weill Cornell Medical College, Houston Methodist Hospital, Houston, TX, USA; e Department of Pharmaceutical Sciences, University of Illinois at Chicago, Chicago, IL, USA; f Center for Translational Research in Oncology, ICESP/HC, Faculdade de Medicina da Universidade de São Paulo and Comprehensive Center for Precision Oncology, Universidade de São Paulo, São Paulo, SP, 01246-000, Brazil

**Keywords:** Nitric oxide synthase 2 (NOS2), COX2-PGE_2_ signaling, Warburg effect, Metabolic reprogramming, Tumor microenvironment (TME)

## Abstract

Nitric oxide synthase 2 (NOS2) and cyclooxygenase 2 (COX2) lie at a critical intersection between inflammation, metabolism, and oncogenic signaling, where they cooperatively promote and establish a Nitric Oxide (NO)-driven Warburg phenotype in advanced cancers. Early work in macrophages established NOS2-derived NO as both a signaling molecule and metabolic stressor that inhibits oxidative phosphorylation (OXPHOS) by targeting iron-sulfur enzymes and respiratory complexes, forcing neighboring cells to rewire metabolism. In human tumors, sustained NOS2 expression in cancer cells and tumor-associated macrophages (TAMs) enforces a Warburg-like state characterized by high glycolytic flux, glutamine dependence, and enhanced NADPH production, supporting proliferation, biosynthesis, and resistance to oxidative stress. At nitrosative-signaling concentrations (≈100-500 nM), NO breaks carbon entry into the TCA cycle at aconitase and pyruvate dehydrogenase, progressively disables dehydrogenase complexes containing dihydrolipoamide dehydrogenase (DLD) and electron-transport complexes (ETCs), and activates hypoxia-inducible factor 1-alpha (HIF-1), phosphoinositide 3-kinase (PI3K)/protein kinase B (Akt), extracellular signal-regulated kinase (ERK)/pyruvate kinase M2 (PKM2)/c-Myc signaling axis, nuclear factor erythroid 2-related factor 2 (Nrf2), and transforming growth factor Beta (TGF-β)/SMAD pathways. These biochemical and signaling effects convert transient glycolytic adaptation into chemically enforced dependency, further stabilized by metabolite-driven inhibition of ten-eleven translocation (TET) and Jumonji demethylases, creating an “epigenetic lock” that maintains oncogenic transcriptional programs. NOS2 and COX2 form a reciprocal feed-forward circuit in which NO, prostaglandin E2 (PGE_2_), interleukin (IL)-6, and IL-8 reinforce one another, driving tumor-promoting inflammation, immunosuppression, angiogenesis, and metastasis while depleting nutrients and acidifying the tumor interstitial fluid. Spatially, NOS2/COX2 niches at the tumor-stroma interface and within immune deserts generate gradients of NO, PGE_2_, oxygen, and metabolites that partition tumors into microdomains with distinct metabolic states, immune composition, and therapeutic vulnerabilities. Integrating these insights with Hanahan's updated hallmarks of cancer, we propose that NOS2-derived NO functions as a node synchronizing deregulated energetics, inflammation, immune evasion, plasticity, and therapy resistance within the tumor microenvironment (TME). Targeting the NOS2-COX2 axis and its downstream NO-iron-epigenetic circuitry may therefore disrupt multiple hallmarks and reveal combinatorial strategies to exploit NO-induced metabolic liabilities in cancer.

## 1 Introduction

Metabolic reprogramming is a defining hallmark of cancer and a critical determinant of both tumor progression and therapeutic response. Among the different microenvironmental factors influencing this process, hypoxia is a major driver that selects for more aggressive cancer phenotypes, enhances cancer stemness, and contributes to resistance against chemotherapy, radiotherapy, and immunotherapy, as well as increased metastatic potential [[1], [2], [3], [4]]. A central metabolic adaptation to this condition is the Warburg effect, first described by Otto Warburg, in which cancer cells preferentially metabolize glucose to lactate despite sufficient oxygen availability, a phenomenon known as aerobic glycolysis [[5], [6], [7]].

The molecular basis of this metabolic shift has been attributed to oncogenic mutations and mitochondrial alterations that activate pro-glycolytic signaling pathways. Mutations in kirsten rat sarcoma GTPase (RAS) or tumor protein 53 (p53) can stimulate HIF-1 stabilization, leading to increased expression of glycolytic enzymes and glucose transporters that sustain anabolic growth [[8], [9], [10]]. Emerging evidence suggests that tumor-derived NO contributes to Warburg-like metabolic reprogramming through suppression of OXPHOS and activation of oncogenic signaling pathways [11,12]. This convergence in metabolic signaling represents a central mechanism driving tumor progression and therapeutic resistance.

Key enzymes in this process include NOS2, which generates NO from l-arginine, and COX2, which produces PGE_2_ from arachidonic acid [13] and participates in a reciprocal, feed-forward inflammatory circuit with NOS2. Increased NOS2 activity and NO production are consistently associated with more aggressive cancers, suggesting that NO serves as a potent mediator of the Warburg effect [11,14].

The Warburg effect confers multiple advantages that support malignancy [15]. These effects include 1) diversion of glycolytic intermediates into biosynthetic pathways for nucleotide, lipid, and amino acid production; 2) lactate accumulation that acidifies the TME and suppresses immune responses; 3) reduced mitochondrial respiration, mitigating reactive oxygen species (ROS)-induced apoptosis; 4) enhanced resistance to anoikis via reduced OXPHOS dependence; and 5) stabilization of HIF-1 and other pro-metastatic transcription factors that link glycolytic reprogramming to angiogenesis, epithelial-mesenchymal transition (EMT), and invasion [16].

**Fig. 1:**
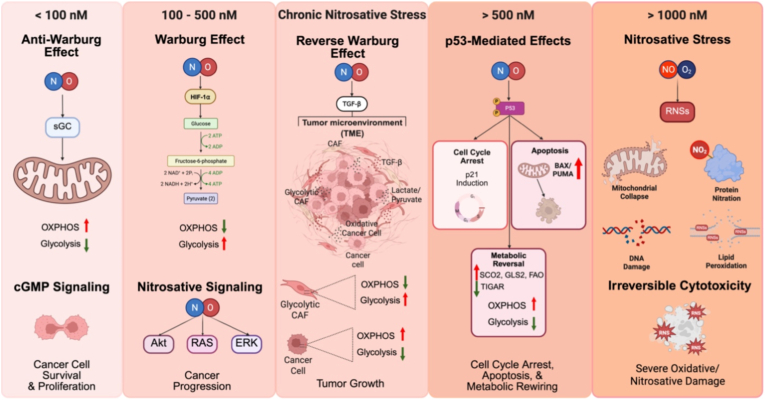
**Nitric oxide concentration-dependent metabolic and signaling effects in cancer.** At very low NO concentrations (<100 nM), activation of soluble guanylate cyclase (sGC) enhances mitochondrial oxidative phosphorylation (OXPHOS) and suppresses glycolysis, producing an anti-Warburg effect that promotes cancer cell survival and proliferation through cGMP signaling. At intermediate NO levels (100–500 nM), NO stabilizes HIF-1α to drive the classical Warburg effect with reduced OXPHOS and increased glycolysis, while nitrosative signaling via Akt, RAS, and ERK supports cancer progression. Similar NO concentrations in the tumor microenvironment (TME) elicit a reverse Warburg effect, whereby cancer-associated fibroblasts (CAFs) become highly glycolytic and fuel oxidative tumor cells with lactate/pyruvate, collectively promoting tumor growth. At higher NO concentrations (>500 nM), p53-dependent signaling induces p21-mediated cell cycle arrest, activation of pro-apoptotic factors (BAX, PUMA), and metabolic reversal characterized by increased OXPHOS and decreased glycolysis, leading to cell cycle arrest, apoptosis, and metabolic rewiring; p53 also exerts inhibitory feedback on NO production, thereby limiting further NO accumulation. At very high NO levels (>1000 nM), formation of reactive nitrogen species (RNS) causes mitochondrial collapse, protein nitration, lipid peroxidation, and DNA damage, resulting in severe nitrosative stress and irreversible cytotoxicity.

Over the past decade, NO has been shown to reproduce many features of hypoxia and aerobic glycolysis by activating oncogenic signaling pathways [17,18]. Quantitative studies in murine tumor models have shown that NO concentrations in the range of ≈100-500 nM can enhance chemoresistance, metastasis, and immunosuppressive activity while concurrently suppressing OXPHOS [13,18]. Building on these observations, preliminary data from our laboratory indicate that NO levels within this range are also achieved in human breast cancer cells at the single-cell level. The convergence of NO signaling and metabolic reprogramming underscores the role of NO as an active driver of the Warburg effect, acting independently of or synergistically with oncogenic mutations. In this work, we examine the contributions of NOS2-derived NO to cancer metabolism, highlighting its integrated roles in aerobic glycolysis, mitochondrial regulation, modulation of the p53 pathway, and remodeling of the TME at the single-cell level (Fig. 1).

## 2 NOS2-derived nitric oxide as a driver of Warburg-like metabolic reprogramming and tumor progression

Early work in the 1980's showed that NOS2-derived NO can suppress cellular respiration by targeting critical enzymes in the tricarboxylic acid (TCA) cycle and diverting glycolytic flux toward alternative metabolic pathways, initially described in macrophages but with clear implications for tumor cells [19]. This persistent NO flux is shown to inhibit the TCA cycle, enhance lactate production, and stabilize a Warburg-like metabolic state that supports proliferation, redox buffering, and cancer survival (Fig. 1) [20,21].

Early work on NOS2 demonstrated that NO functions not only as a signaling molecule but also as a significant metabolic stressor in cancer cells. In classic studies using murine macrophages, inflammatory activation induced an l-arginine-dependent peak of NO that inhibits mitochondrial respiration in nearby murine lymphocytic leukemia cells, slowing their proliferation and, at higher levels, killing them completely [[22], [23], [24]].

At the biochemical level, this nitrosative NO flux (≈100-500 nM) selectively reacts with iron-sulfur cluster-containing enzymes, including aconitase 2 (ACO2) and components of complexes I and II of the ETC, disrupting OXPHOS and forcing tumor cells to rewire their metabolism towards glycolysis, thus promoting a Warburg-like phenotype (Fig. 1). At the same time, these Nos2-expressing murine macrophages accumulated nitrite (NO_2_^−^) and nitrate (NO_3_^-^) and promoted the nitrosation of secondary amines, demonstrating that chronic inflammatory activation can generate sufficient NO and dinitrogen trioxide (N_2_O_3_) to form potentially carcinogenic nitrosamines [[25], [26], [27], [28]]. This combination of high-flux NO, OXPHOS inhibition, and nitrosative chemistry sets activated murine macrophages apart from endothelial cells, in which NO levels are much lower and primarily control vasodilation. Importantly, at lower NO fluxes (<100 nM), NO preferentially signals through soluble guanylate cyclase (sGC) and cyclic guanosine monophosphate (cGMP)-dependent pathways, promoting mitochondrial biogenesis, preservation of OXPHOS, and suppression of glycolysis, establishing an “anti-Warburg” metabolic shift that contrasts with the glycolytic reprogramming observed at nitrosative signaling concentrations (Fig. 1) [29]. Together, these findings introduced a nuanced concept that remains highly relevant for advanced cancers: NOS2-derived NO in the TME can both restrain tumor growth by disabling mitochondrial metabolism and, under chronic inflammatory conditions, contribute to DNA damage and selection pressures that influence long-term cancer progression.

As NO flux increases beyond the classical nitrosative signaling window (≈100-500 nM), its biological consequences shift from metabolic enforcement of glycolysis to activation of tumor-suppressive stress checkpoints. At concentrations exceeding ∼500 nM, NO-induced genotoxic and mitochondrial stress can stabilize and activate p53, triggering a coordinated cytotoxic program characterized by cyclin-dependent kinase inhibitor 1A (p21)-mediated cell cycle arrest, induction of pro-apoptotic mediators such as Bcl-2-associated X protein (BAX) and p53 upregulated modulator of apoptosis (PUMA), and partial metabolic reversal through upregulation of the synthesis of cytochrome *c* oxidase 2 (SCO2), glutaminase 2 (GLS2), and fatty acid oxidation pathways. In tumors retaining wild-type p53, this threshold response can dampen NOS2 and COX2 expression, restrain glycolytic commitment, and transiently restore oxidative metabolism, thereby functioning as a protective checkpoint against uncontrolled nitrosative signaling. However, in p53-mutant or dysfunctional tumors, this regulatory mechanism is lost, allowing sustained NOS2/COX2 activity and continued metabolic rewiring (Fig. 1).

When NO levels increase further (>1000 nM), the balance shifts toward nitrosative stress and irreversible cytotoxicity, driven by excessive formation of reactive nitrogen species (RNS), mitochondrial collapse, DNA damage, protein nitration, and lipid peroxidation. At this stage, cellular injury becomes largely p53-independent and reflects chemical oxidative/nitrative catastrophe rather than regulated metabolic signaling (Fig. 1). However, such extreme NO flux is unlikely to be uniform across tumors. Instead, fluctuating, spatially heterogeneous NO gradients within the TME more commonly sustain intermediate nitrosative signaling levels, favoring metabolic adaptation over cytotoxicity. Under these chronic but sublethal conditions, NO does not simply kill tumor cells; rather, it exerts selective pressure that reshapes metabolic circuitry and promotes the survival of clones that tolerate persistent mitochondrial stress [13].

In advanced human tumors, this foundational work has expanded into a broader concept in which NOS2 acts as a dynamic modulator of cancer cell metabolism, shaping outcome and progression in a context-dependent manner [12]. Clinical and experimental studies in breast, ovarian, colorectal, and hepatobiliary cancers show that sustained NOS2 expression in tumor cells and tumor-associated macrophages (TAMs) drives metabolic programs that support a Warburg-like phenotype, characterized by high glycolytic flux, glutamine dependence, and increased NADPH production, which together sustain rapid growth, biosynthesis, and oxidative stress resistance in cancer cells [20,[30], [31], [32], [33]].

In ovarian cancer, for example, NOS2-derived NO promotes glycolysis by inducing PKM2 nuclear translocation via epidermal growth factor receptor (EGFR)/extracellular signal-regulated kinase 2 (ERK2) signaling, enhancing ATP and NADPH generation, and correlating with aggressive tumor behavior and poor survival [11,31]. Similar NOS2-associated metabolic signatures, including altered TCA cycle flux, activation of the pentose phosphate pathway, and enhanced lipid and nucleotide synthesis, have been reported in advanced breast and colorectal cancers, where high NOS2 expression often associates with advanced stages, metastasis, chemoresistance, and shorter disease-free survival [20,30,32].

Emerging data in hepatoblastoma, triple-negative breast cancer, and other advanced malignancies indicate that high NOS2 expression is coupled to distinct metabolic states that interface with immune evasion, such as reduced CD8^+^ T cell infiltration and enrichment of immunosuppressive myeloid populations, linking NO-driven metabolic rewiring not only to tumor-intrinsic fitness but also to a more permissive TME [20,33,34].

Together, these observations position NOS2-derived NO as a concentration-dependent metabolic regulator that shifts from enforcing a Warburg-like phenotype to activating stress checkpoints and, at extreme levels, inducing nitrosative collapse.

## 3 Linking NO flux to OXPHOS suppression and oncogenic signaling in tumor cells at the single-cell level

Prior studies using murine 4T1 breast cancer cells indicate that Nos2 induction in these cells can generate NO levels comparable to those observed in stimulated murine macrophages, with NO_2_^−^ accumulation scaling with the proportion of Nos2-expressing cells [30,35,36]. In murine macrophage models, 40-85% of cells can express Nos2 under inflammatory stimulation, whereas in human cancer cell lines exposed to similar stimuli, only about 3-5% of cells become NOS2^+^; nevertheless, NO_2_^−^ production per NOS2^+^ cell suggests that enzyme activity at the single-cell level is comparable between species [14,30,35,36]. Importantly, NO flux within this range has previously been shown to activate oncogenic signaling, particularly through HIF-1α stabilization and activation of the Ras/ERK and PI3K/Akt pathways. This indicates that even a small fraction of NOS2^+^ tumor cells could have a disproportionately strong signaling impact within the TME [13].

**Fig. 2:**
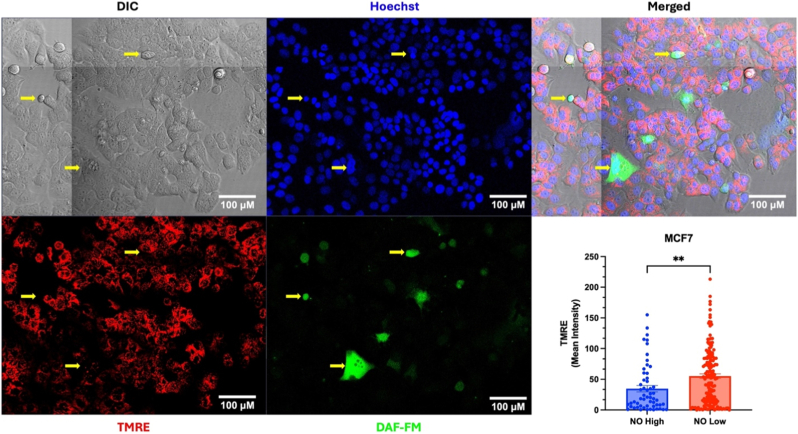
**Single-cell inverse relationship between NO levels and mitochondrial membrane potential in MCF7 cells.** MCF7 cells were co-stained with Hoechst (nuclei, blue), TMRE (mitochondrial membrane potential, red), and DAF-FM (intracellular NO, green) and imaged by fluorescence microscopy. Yellow arrows highlight DAF-FM-high cells that exhibit low or undetectable TMRE fluorescence, indicating loss of mitochondrial membrane potential in high-NO cells. The merged image (right, top row) overlays DIC, Hoechst, TMRE, and DAF-FM channels to visualize the spatial relationship between NO production and mitochondrial depolarization. The scatter/box plot (bottom right) shows TMRE mean intensity quantified in individual cells stratified as NO High (high DAF-FM) or NO Low (low DAF-FM), demonstrating significantly reduced TMRE signal in NO-high cells, consistent with NO-dependent mitochondrial impairment. Statistical significance was assessed by unpaired two-tailed Student's t-test (∗∗P < 0.01).

To determine how increased NOS2 activity affects metabolism in individual tumor cells and support the current review, we used high-resolution fluorescence imaging in human MCF7 breast cancer cells, which exhibit elevated basal NOS2 expression. Cells were co-stained with the intracellular NO probe DAF-FM, a fluorescent dye that reacts with NO-derived species to report intracellular NO levels, and the mitochondrial membrane potential dye TMRE, a potentiometric dye that accumulates in active mitochondria in proportion to membrane potential. This approach allowed us to measure intracellular NO and OXPHOS simultaneously at the single-cell level. We observed that cells with high DAF-FM fluorescence consistently showed reduced TMRE fluorescence, indicating an inverse relationship between intracellular NO and mitochondrial membrane potential in MCF7 cells (Fig. 2).

Preliminary calibration experiments with the NO donor DETANO indicate that the TMRE reduction occurs within an NO range of approximately 100-400 nM (unpublished data), a window sufficient to induce metabolic reprogramming via HIF-1α stabilization and to engage major oncogenic mediators such as Ras, ERK, and Akt, as previously mentioned (Fig. 1). These observations support a model in which NOS2-derived NO at physiologically relevant concentrations simultaneously suppresses OXPHOS and potentiates oncogenic signaling, thereby linking the NO-driven Warburg-like phenotype to pro-tumor signaling pathways in human breast cancer cells.

Taken together, our findings align with the current literature demonstrating that NO acts as a potent regulator of mitochondrial metabolism, where sustained NOS2-derived NO suppresses OXPHOS through inhibition of ETC components and TCA cycle enzymes, thereby reinforcing Warburg-like metabolic reprogramming in cancer cells [[37], [38], [39]].

## 4 Nitrosative biochemistry underlying the nitric oxide-induced Warburg shift

**Fig. 3:**
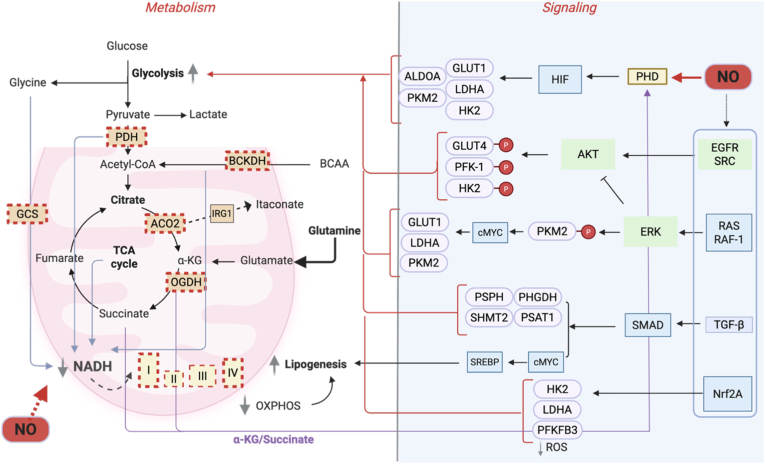
**Metabolic and Signaling Map of NO**. The diagram illustrates the dual role of NO in modulating cellular function, bridging the gap between metabolic flux and signaling pathways. At concentrations of 100-500 nM, NO acts as a master regulator by inhibiting key mitochondrial enzymes and activating signaling cascades that promote a pro-proliferative, glycolytic phenotype. *Metabolic Impact:* NO induces a metabolic shift by inhibiting several mitochondrial enzymes (indicated by red dashed boxes), including PDH, ACO2, OGDH and other dihydrolipoamide dehydrogenase subunit-containing enzymes (BCKDH, GCS), and the complexes of the electron transport chain (ETC) (complexes I-IV). This results in decreased OXPHOS and a compensatory increase in glycolysis and lipogenesis. The accumulation of TCA cycle intermediates like ⍺-KG and succinate further facilitates signaling and epigenetic changes. *Signaling Cascades:* NO triggers multiple pathways (e.g., HIF, AKT, ERK) that upregulate glucose transporters (GLUTs) and glycolytic enzymes (e.g., HK2, PKM2). It also modulates transcription factors like cMYC and SREBP to support biosynthetic requirements and antioxidant defenses (via Nrf2A). ⍺-KG: ⍺-Ketoglutarate; ACO2: Aconitase 2; BCAA: Branched-Chain Amino Acids; BCKDH: Branched-Chain -Keto Acid Dehydrogenase; GCS: Glycine Cleavage System; NADH: Nicotinamide Adenine Dinucleotide (reduced); OGDH: -Ketoglutarate Dehydrogenase; OXPHOS: Oxidative Phosphorylation; PDH: Pyruvate Dehydrogenase; TCA cycle: Tricarboxylic Acid Cycle (Krebs Cycle); AKT: Protein Kinase B;, ALDOA: Aldolase A; cMYC: Myelocytomatomatosis oncogene; EGFR: Epidermal Growth Factor Receptor; ERK: Extracellular Signal-Regulated Kinase; GLUT1/4: Glucose Transporter 1/4; HIF: Hypoxia-Inducible Factor; HK2: Hexokinase 2; LDHA: Lactate Dehydrogenase A; Nrf2A: Nuclear Factor Erythroid 2-Related Factor 2; PFK-1/PFKFB3: Phosphofructokinase isoforms; PHD: Prolyl Hydroxylase; PKM2: Pyruvate Kinase M2; ROS: Reactive Oxygen Species; SREBP: Sterol Regulatory Element-Binding Protein.

Determining how much NO can be produced at the single-cell level provides an opportunity to link specific NO chemistry to the modification of key proteins that control metabolic reprogramming. In the absence of NO or under normoxic conditions, glucose is metabolized through classical glycolysis to pyruvate, which is converted to acetyl-CoA and enters the TCA cycle to generate NADH for mitochondrial respiration [40]. However, in addition to its well-characterized NO-driven reversible inhibition of complex IV (cytochrome *c* oxidase) through competition with oxygen at the heme-copper center, at nitrosative signaling levels (≈100-500 nM) NO can act simultaneously at several critical sites within the mitochondrial metabolic machinery, including iron-sulfur cluster-containing enzymes of the TCA cycle (ACO2), the pyruvate dehydrogenase (PDH) complex, and multiple components of the ETC. At these sites, NO exerts its effects either by directly binding to redox-active metal centers (heme and non-heme iron) or by promoting S-nitrosation and other thiol modifications in hydrophobic protein environments, thereby altering both catalytic activity and downstream signaling [38,41,42] (Fig. 3).

### 4.1 Direct mitochondrial targets - TCA cycle and ETC

At the level of the TCA cycle and mitochondrial respiration, NOS2-derived NO was recognized in the late 1980s and early 1990s as a potent inhibitor of cellular respiration [24]. As previously mentioned, one major direct NO target is the iron-sulfur (Fe-S) clusters in enzymes such as ACO2, whose inactivation interrupts the TCA cycle and restricts NADH supply to the respiratory chain (Fig. 3) [43]. In parallel, because NO and O_2_ compete for complex IV, only low micromolar to submicromolar O_2_ (≈10-450 nM) is required for NO to inhibit respiration, meaning that tissue oxygen tensions typical of tumors (≈1-3% O_2_) favor this interaction [44]. Inhibition at complex IV not only slows electron flow but also increases NO reaction with superoxide generated at complex III, forming peroxynitrite (ONOO^−^) and, subsequently, N_2_O_3_, which can nitrosate critical thiols or be converted to NO_2_^−^.

At nitrosative signaling levels, however, NO also activates nuclear factor kappa-light-chain-enhancer of activated B cells (NF-κB), inducing manganese superoxide dismutase (MnSOD) and limiting NO-superoxide reactions, thereby preserving mitochondrial integrity [45]. Additional work indicates that thiol nitrosation at complexes I and II can restrict specific NADH entry points from different segments of the TCA cycle, providing another layer of metabolic control at these NO levels [46]. Collectively, these mitochondrial targets reduce electron flow, diminish NADH oxidation, alter membrane potential, and restrict OXPHOS, thereby shifting cellular metabolism toward aerobic glycolysis. Mild mitochondrial depolarization under these conditions may even protect against ischemia-reperfusion-like injury associated with rapidly growing tumors [47]. Thus, at appropriately tuned nitrosative signaling levels, NO can simultaneously restrain oxidative metabolism, promote aerobic glycolysis, and paradoxically help maintain mitochondrial function, thereby supporting tumor cell survival and adaptation.

## 5 Chemistry of nitric oxide at the nitrosative signaling level and the NO-induced Warburg shift

### 5.1 Aconitase 2 (ACO2)

At nitrosative signaling levels (≈100-500 nM), NO imposes direct, concentration-dependent control over key metabolic enzymes, with effects that extend both within NO-producing cells and to neighboring cells in the TME. In mitochondria, NO selectively inhibits ACO2 by oxidizing its iron-sulfur cluster [4Fe-4S], leading to iron release, loss of catalytic activity, and a disruption of the TCA cycle that drives citrate accumulation and constrains itaconate production through immune-responsive gene 1 (IRG1) [22,[48], [49], [50]] (Fig. 3). The lipophilic nature of NO and its enrichment in mitochondrial membranes favor ACO2 over cytosolic ACO1 as a target, establishing ACO2 inhibition as a metabolic rheostat in TAMs. ACO2 inhibition prevents carbon flow from citrate to α-ketoglutarate (α-KG) and limits *cis*-aconitate availability for itaconate synthesis, creating a barrier that metabolically distinguishes high-NO TAMs from cells with intact aconitase function [50].

### 5.2 Pyruvate dehydrogenase (PDH) complex

Beyond ACO2, NO also targets the PDH complex (Fig. 3) via a lipoate-dependent mechanism that generates nitroxyl (HNO), a more reduced and highly reactive NO species [51,52]. Lipoyl groups on the PDH-E2 subunit act as both substrates and catalysts for HNO formation, as NO attacks lipoyl-SH and the neighboring thiol converts NO to HNO in close proximity to the complex. Locally generated HNO irreversibly modifies the DLD/E3 subunit at key cysteines, particularly Cys484 and Cys477, forming sulfinamide adducts that disrupt homodimer formation and abolish enzymatic activity. Proteomic analyses in NO-producing macrophages show accumulation of PDH-E2 peptides bearing reduced- and acetyl-lipoate, consistent with a stalled PDH machine, and a unique acetyl-nitroso-lipoate species that appears only in the presence of NO. Structural modeling indicates that sulfinamide formation at Cys484 destabilizes critical interchain contacts within DLD, further explaining the loss of PDH function [38].

### 5.3 Other DLD-containing enzyme complexes

This HNO-driven sulfinamide chemistry extends beyond PDH to other DLD-containing complexes, including α-ketoglutarate dehydrogenase (OGDH), branched-chain keto-acid dehydrogenase (BCKDH), and the glycine cleavage system (GCS) [53]. Because these dehydrogenases share the DLD subunit, a single HNO-generating process can simultaneously disrupt multiple biosynthetic and anaplerotic routes, forcing cells to rely on alternative pathways such as glutaminolysis, pyruvate carboxylation, and the gamma-aminobutyric acid (GABA) shunt to sustain TCA intermediates (Fig. 3). Unlike reversible S-nitrosation, sulfinamide formation is essentially irreversible, so the resulting mitochondrial dysfunction and metabolic rewiring persist even after NO levels fall, effectively establishing an altered metabolic state [38].

In TAMs, the combined inhibition of ACO2 and PDH yields a characteristic metabolic program with suppressed oxidative metabolism, enhanced glycolysis, and re-channeled TCA flux driven by glutaminolysis. After NO-mediated blockade of glucose-derived entry into the TCA cycle, TAMs show reduced glucose carbon flux and a 2-3-fold increase in glutamine uptake and metabolism [50]. Glutamine-dependent anaplerosis maintains α-KG and other intermediates needed for biosynthesis, including collagen/proline production for extracellular matrix remodeling and citrate for lipid synthesis. Shifts in metabolite ratios feed back as signaling cues: reduced α-KG alters α-KG-dependent dioxygenases TET enzymes and histone demethylases, promoting epigenetic changes that stabilize immunosuppressive programs, while succinate accumulation, largely glutamine-derived in NO-exposed macrophages, stabilizes HIF-1α by inhibiting prolyl hydroxylases (PHDs), driving a pseudo-hypoxic transcriptional state [[54], [55], [56], [57], [58], [59]].

### 5.4 Mitochondrial respiration (site I/II)

NO-mediated inhibition of the ETC complexes adds a second layer of mitochondrial control. Although complex IV is reversibly inhibited at nanomolar NO levels via competition with O_2_ at the a_3_-CuB center, sustained NO exposure, together with substrate limitation from ACO2/PDH inhibition, drives irreversible loss of complexes I-IV through an active/deactive (A/D) transition of complex I [[60], [61], [62]] (Fig. 3). Under low NADH conditions, complex I accumulates in the deactive (D) form, which exposes Cys39 in the NADH dehydrogenase subunit 3 (ND3) to modification by NO-derived species. S-nitrosation of Cys39 in the D form blocks reactivation by NADH and marks the complex for degradation, whereas the A form is relatively resistant [50]. This mechanism explains the pronounced late loss of ETC proteins during proinflammatory polarization, where diminished NADH/NAD^+^ ratios favor D-form accumulation, post-translational modification, and proteolysis [41].

### 5.5 Site III, MnSOD versus NO consumption

At complex III, where electron leak generates superoxide, the interplay between NO and mitochondrial ROS becomes a critical determinant of whether NO remains a signaling molecule or is diverted into damaging RNS. Superoxide from the ETC can be rapidly dismutated by mitochondrial MnSOD, limiting downstream radical chemistry, but if superoxide is not efficiently cleared, it reacts with NO at diffusion-limited rates to form ONOO^−^ and, promptly, secondary nitrogen oxides such as nitrogen dioxide (NO_2_) and N_2_O_3_ [50,[63], [64], [65]]. Functionally, this creates a mitochondrial “fate decision” for NO: high MnSOD activity diverts superoxide away from reacting with NO, limiting ONOO^−^ and other RNS formation, preserving NO within its signaling range, whereas high superoxide flux at complex III promotes NO consumption into NO_2_/N_2_O_3_ and shifts from reversible signaling to oxidative/nitrosative stress that can damage Fe-S enzymes and ETC components [[66], [67], [68]]. In this way, complex III superoxide levels and MnSOD buffering act as a gate, determining whether NO exerts regulated respiratory control or is converted into secondary species that contribute to ETC collapse and broader redox remodeling.

### 5.6 Impact on sugar metabolism

At higher NO and RNS levels (≈100-500 nM), nitrosative signaling extends to glycolytic enzymes, such as glyceraldehyde-3-phosphate dehydrogenase (GAPDH), which can be inhibited by S-nitrosation and NAD^+^ depletion, further uncoupling glycolytic flux from mitochondrial oxidation [69]. A central consequence of NO-induced breaks at ACO2 and PDH is that they do more than passively suppress mitochondrial carbon oxidation; they actively enforce glycolytic commitment as both a compensatory ATP source and a means to redirect carbon into cytosolic biosynthesis. In inflammatory macrophages, endogenous NO is necessary and sufficient to maintain a glycolytic state by limiting pyruvate entry into the TCA cycle and sustaining high glycolytic flux even when respiration is curtailed, while IL-10 acts as a rheostat that dampens NOS2 and modulates how rigidly cells are committed to glycolysis [43].

Thus, sugar metabolism becomes an integrated, NO-imposed architecture that couples bioenergetic rescue (ATP from glycolysis) with redox capacity (NADPH from ancillary pathways) and effector function (cytokine and mediator production) [50,[70], [71], [72], [73]]. Functionally, this has distinct temporal consequences in tumors: early glycolytic reprogramming supports plasticity and survival [74], whereas late, NO-driven glycolysis under chronic nitrosative stress favors malignant selection of clones that withstand high redox stress, resist apoptosis, and thrive in acidic, hypoxic niches. In this late phase, glycolysis aligns with cancer stem cell traits, therapy resistance, and immune exclusion, whereas the early phase retains some reversibility. In NO-producing tumor cells, glycolysis thus evolves from a flexible adaptation into a chemically enforced dependency, with NO acting as the switch that converts metabolic plasticity into a permanent metabolic fate [20].

### 5.7 Lipid storage and consumption

While not always massively fueling de novo lipogenesis, citrate accumulation downstream of ACO2 inhibition serves as a marker of the depth of TCA disruption and can be exported to shape the extracellular metabolic milieu. Thus, NO-mediated suppression of mitochondrial metabolism also exerts a direct effect on lipid metabolism. Rather than favoring citrate-driven fatty acid synthesis alone, inflammatory activation under high NO flux reduces fatty acid oxidation (including via decreased carnitine palmitoyltransferase 1 (CPT1)-dependent import), diverting fatty acids toward neutral lipid storage. Multiple studies show increased incorporation of fatty acids into triglycerides, upregulation of glycerolipid synthesis enzymes such as glycerol-3-phosphate acyltransferase 3 (GPAT3) and diacylglycerol O-acyltransferase 2 (DGAT2), and reduced triglyceride lipolysis, yielding a lipid droplet-rich phenotype. Notably, this can be NO-imposed: treatment of resting macrophages with the NO donor DETANO is sufficient to induce lipid droplet formation, consistent with a model in which NO suppresses respiration and secondarily blocks fatty acid oxidation, driving neutral lipid accumulation from imported lipids [[75], [76], [77], [78]]. In this context, lipid droplets become a visible readout of the same NO-driven mitochondrial constraints that enforce glycolysis, linking nitrosative/inflammatory signaling to a durable shift in lipid storage versus consumption.

In addition, nitrosative signaling may also regulate lipid anabolic pathways through post-translational modification of metabolic enzymes. In this context, S-nitrosation of fatty acid synthase (FASN) has been proposed as a mechanism through which NO directly modulates lipid biosynthesis under chronic inflammatory conditions [79].

Taken together, these layers of NO biochemistry position nitrosative signaling as a master switch for the Warburg phenotype in cancer. By simultaneously breaking carbon entry into the TCA cycle at ACO2 and PDH level, disabling multiple DLD-containing dehydrogenases, and progressively disrupting ETC capacity at complexes I-IV, NO forces cells to transition from oxidative ATP production to a glutamine-supported, glycolysis-dependent metabolism that is reinforced at the epigenetic and transcriptional levels via α-KG/succinate, HIF-1α, and NF-κB. In parallel, the MnSOD-superoxide “gate” and direct nitrosation of glycolytic and lipid-handling enzymes determine whether NO remains a reversible respiratory regulator or drives a more permanent shift toward nitrosative stress, glycolytic commitment, and lipid droplet accumulation (Fig. 3). Across tumor and myeloid compartments, this concerted reprogramming couples energy production, redox buffering, and biosynthesis to NOS2 activity, establishing NO not as a passive byproduct of inflammation but as a central signaling molecule capable of installing and stabilizing a Warburg-like metabolic state in cancer cells.

## 6 Complementary effects at nitrosative signaling levels to the nitric oxide-induced Warburg shift

### 6.1 Hypoxia-inducible factor 1-⍺ (HIF-1α)

At nitrosative signaling levels, NO stabilizes HIF-1α primarily by inhibiting PHDs, preventing HIF-1α degradation and allowing its accumulation in the nucleus [80]. Stabilized HIF-1α binds hypoxia response elements (HREs) and drives expression of genes that reinforce the Warburg phenotype, including glucose transporters (GLUTs) such as GLUT1 and a broad panel of glycolytic enzymes (e.g., hexokinase, aldolase, GAPDH, PKM2, lactate dehydrogenase A (LDHA)), thereby promoting high glucose uptake and preferential conversion of pyruvate to lactate even in the presence of oxygen (aerobic glycolysis) [81] (Fig. 3). Lactate itself, together with PKM2, feeds back positively into this circuit: lactate can stabilize HIF-1α, and PKM2 can interact with and further sustain HIF-1α and STAT3 signaling, amplifying transcriptional programs that support glycolysis, survival, and inflammatory signaling [82,83]. Through these interconnect loops, nitrosative NO signaling and HIF-1α create a self-reinforcing axis that anchors the metabolic shift initiated by direct NO-driven inhibition of mitochondrial enzymes.

### 6.2 Phosphoinositide 3-kinase (PI3k)/Akt

In parallel, nitrosative NO levels (≈100-500 nM) also activate the PI3K/Akt pathway. Nitrosation of EGFR and proto-oncogene tyrosine-protein kinase (Src) promotes PI3K activation and, together with NO-mediated inhibition of the tumor suppressor phosphatase and tensin homolog (PTEN), leads to sustained Akt phosphorylation [18,84]. Activated Akt enhances glucose uptake by phosphorylating TBC1 domain family member 1 (TBC1D1), thereby facilitating GLUT4 translocation to the plasma membrane and increasing glucose import [85]. Akt signaling also phosphorylates and activates key glycolytic control points, including hexokinase and phosphofructokinase, thereby accelerating glycolytic flux while simultaneously driving protein and lipid synthesis required for biomass accumulation and cell division [86] (Fig. 3). In this way, NO-dependent Akt activation complements HIF-1α by ensuring that the transcriptional drive for glycolysis is matched by post-translational activation of glucose uptake and glycolytic enzymes.

### 6.3 Extracellular signal-regulated kinase (ERK)

NO-dependent signaling further converges on ERK via nitrosation and activation of RAS [87]. Activated ERK phosphorylates PKM2, promoting its nuclear translocation, where nuclear PKM2 functions as a protein kinase and transcriptional co-activator for genes that reinforce glycolysis and proliferation [88]. Nuclear PKM2 enhances c-Myc activity, a master regulator of aerobic glycolysis, thereby increasing expression of PKM2 itself, GLUT1, and LDHA, establishing a feed-forward loop that keeps cells in high glycolytic flux [89]. ERK also suppresses mitochondrial respiration indirectly by inhibiting AMPK. ERK-P phosphorylates and inhibits liver kinase B1 (LKB1), a key AMP-activated protein kinase (AMPK) activator, thereby leaning the balance further toward glycolysis and away from oxidative metabolism [90]. Together with HIF-1α and Akt, ERK-PKM2-*c*-Myc signaling completes a tightly coupled transcriptional and post-translational network through which NO converts transient glycolytic adaptation into a robust oncogenic program (Fig. 3).

### 6.4 Nuclear factor erythroid 2-related factor 2 (NrF2a)

Although Nrf2 does not directly induce glycolytic enzyme expression, its activation at nitrosative signaling levels provides an essential antioxidant scaffold that sustains the Warburg effect. By upregulating genes involved in glutathione synthesis, ROS detoxification, and NADPH regeneration, Nrf2 enables NO-exposed and glycolysis-addicted tumor cells to tolerate the heightened redox burden associated with rapid glucose metabolism and impaired mitochondrial respiration [91] (Fig. 3). In this sense, Nrf2 does not drive glycolysis per se but shields NO-driven, HIF/Akt/ERK-dependent metabolic rewiring from oxidative collapse, allowing Warburg metabolism to persist under chronic stress.

### 6.5 Transforming growth factor Beta (TGF-β)/SMAD

Nitrosative levels of NO facilitate TGF-β activation, in part by converting latent TGF-β to its active form, which then engages SMAD signaling [92]. Activated TGF-β/SMAD pathways reinforce the Warburg effect by promoting transcription of key glycolytic and anabolic genes, including GLUT1, 6-phosphofructo-2-kinase/fructose-2,6-biphosphatase 3 (PFKFB3), and PKM2, often through synergistic interactions with c-Myc [[93], [94], [95], [96]] (Fig. 3). Beyond glycolysis, the SMAD-*c*-Myc axis can reshape amino acid and lipid metabolism, for example, by upregulating enzymes in glycine-serine biosynthesis and modulating fatty acid oxidation, thereby supporting nucleotide production and membrane biogenesis in proliferating cancer cells. Through these actions, NO-enabled TGF-β/SMAD signaling helps couple metabolic rewiring to a more immunosuppressive, pro-fibrotic TME that is characteristic of advanced cancers.

### 6.6 Tumor protein 53 (p53)

As discussed above, p53 represents a major negative regulator of NOS2/COX2-driven nitrosative signaling. Wild-type p53 acts as a metabolic gatekeeper that directly opposes the NO-induced Warburg shift through multiple complementary mechanisms [13].

p53 induces a group of genes that directly antagonize Warburg metabolism: SCO2 and other mitochondrial components support complex IV function and restore oxidative phosphorylation; GLS2 channels glutamine into the TCA cycle to bolster OXPHOS; and TP53-induced glycolysis and apoptosis regulator (TIGAR) reduces fructose-2,6-bisphosphate levels, thereby decreasing PFK1 activity and slowing glycolysis [16,97]. Simultaneously, stabilized wild-type p53 favors fatty acid β-oxidation, partially reversing the lipid-anabolic bias created by NO-HIF-Akt signaling [97]. NO-activated HIF-1α and Akt can increase FASN, both by rewiring TCA-derived citrate export and by transcriptional induction, thereby aligning aerobic glycolysis with enhanced lipogenesis [98].

In tumors with intact p53, these coordinated responses can both reduce NOS2 expression and restore OXPHOS, but effective reversal requires NO levels to drop sufficiently to restore the TCA cycle [97]. In many aggressive cancers, however, p53 is mutated or inactivated, allowing NO-driven pathways to dominate [99]. This loss of p53 function eliminates the negative feedback loop that normally restricts NOS2 and COX2 induction, creating a self-reinforcing circuit in which chronic NO production drives metabolic reprogramming, immune suppression, and therapy resistance without regulatory constraint [100].

### 6.7 NOS2 and arginine metabolism

**Fig. 4:**
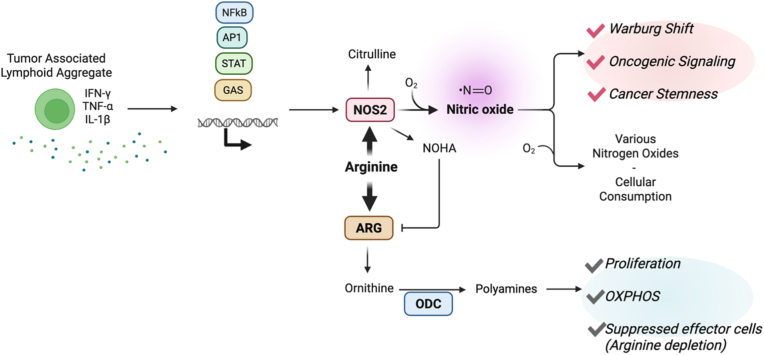
**NOS2–arginine metabolism axis in the tumor microenvironment.** Inflammatory cytokines (IFN-γ, TNF-α, IL-1β) activate nuclear factor kappa-light-chain-enhancer of activated B cells (NF-κB), activator protein 1 (AP-1), and STAT signaling to induce NOS2 expression. NOS2 converts arginine to nitric oxide (NO) and citrulline, promoting oncogenic signaling, the Warburg shift, and cancer stemness. In parallel, arginase (ARG) depletes arginine by converting it to ornithine, which, via ornithine decarboxylase (ODC), supports proliferation and oxidative phosphorylation (OXPHOS). Arginine depletion suppresses immune cell function, particularly T cell activation and effector responses.

The metabolic rewiring imposed by p53 loss converges with arginine metabolism at a crucial regulatory node where NOS2 activity and the Warburg effect intersect to control both tumor cell energetics and immune responses. Arginine can be metabolized by NOS2 to produce NO and citrulline, or by arginase I to generate ornithine, which feeds polyamine synthesis via ornithine decarboxylase [101,102] (Fig. 4). Although NOS2 and arginase differ markedly in their kinetic properties, NOS2 has a much lower Michaelis constant (Km) for arginine but a far lower maximal velocity (Vmax) than arginase, the combination of high catalytic capacity for arginase and high substrate affinity for NOS2 means that, under physiological intracellular arginine concentrations (≈100-800 μM), both enzymes can metabolize substantial fractions of the arginine pool, creating a context-dependent balance between NO production and ornithine/polyamine synthesis [103,104]. However, the NOS2 intermediate N-hydroxy-l-arginine (NOHA) is a potent arginase inhibitor, creating a feedback loop that favors continued NO production once NOS2 is active, thereby favoring a Warburg-like, NOS2-high state. Conversely, when NOS2 is low, and arginase predominates, arginine is diverted into polyamines that support proliferation and, through local arginine depletion, can suppress anti-tumor immune responses [105,106] (Fig. 4).

In macrophages, this balance underlies the contrast between M1-like, NOS2-high, NO-producing cells with Warburg effect and immunosuppressive features, and M2-like, arginase-high cells that emphasize tissue repair but also contribute to tumor progression [38,41,50]. In tumor cells, NO levels sufficient to induce the Warburg effect are closely associated with an invasive and immunosuppressive microenvironment. In contrast, arginase-dominant tumors may maintain more “classical” oxidative metabolism, support rapid proliferation, and deplete regional arginine in ways that impair T cell responses. Extracellular polyamines released by arginase-high tumors can directly suppress effector CD8^+^ T cell activation and function. In contrast, inhibition of polyamine synthesis has been linked to preservation of memory-like and tissue-resident memory T cell pools [30,[107], [108], [109]].

Across these interconnected pathways, nitrosative signaling levels of NO emerge as the central point that links them together: simultaneously stabilizing HIF-1α, activating Akt and ERK, engaging Nrf2 and TGF-β/SMAD, antagonizing p53, and reshaping arginine metabolism in favor of NOS2 and polyamine-mediated immunosuppression. Together with the direct mitochondrial and enzymatic targets outlined in topic 5, this signaling network converts NO from a transient stress response into a key facilitator and stabilizer of the Warburg effect in cancer and immune cells. Thus, NO connects metabolic reprogramming with stemness, therapy resistance, and immune evasion in advanced cancers.

## 7 Nitric oxide, iron complexes, and the Warburg effect: an epigenetic and metabolic network in cancer biology

The intricate relationship between NO and iron represents one of the most fundamental chemical interactions in biological systems, with profound implications for cellular metabolism and gene regulation. This relationship becomes particularly complex when considering the concentration-dependent and temporal nature of NO signaling, in which distinct threshold concentrations regulate distinct cellular proteins and pathways (Fig. 1). Iron complexes represent a principal chemical target of NO, establishing a reciprocal regulatory mechanism that underlies numerous pathological processes, particularly in cancer [110,111]. This relationship becomes increasingly complex when considered alongside the metabolic reprogramming characteristic of cancer cells, specifically the Warburg effect, and its downstream epigenetic consequences.

At the nitrosative signaling levels, iron complexes are among the principal targets of NO. Beyond the well-characterized heme interactions occurring at the cGMP level, NO also inhibits non-heme iron complexes, including Fe-S proteins such as ACO2 and PHDs, as previously discussed. The interaction between NO and the chelatable iron pool (CIP) results in the quantitative conversion of cellular iron into paramagnetic dinitrosyliron complexes (DNICs) with thiol-containing ligands [112]. These DNICs represent the most abundant NO-derived cellular adducts, with concentrations of 45-90 μM (900 pmol/mg protein) in activated cells, far exceeding those of other NO-derived species, such as S-nitrosothiols. DNICs form rapidly upon NO exposure (within minutes) and exhibit remarkable stability with a half-life of approximately 80 min after NO removal, remaining detectable for over 24 h. This persistence allows DNICs to function as a "NO capacitor," extending NO-like bioactivity long after enzymatic NO synthesis has ceased [113,114].

### 7.1 Iron-sulfur proteins and the NO-induced Warburg effect

**Fig. 5:**
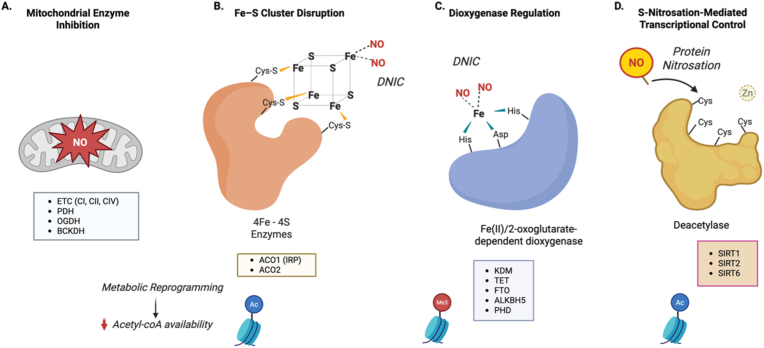
**NO-mediated regulation of iron-sulfur proteins, iron-dependent enzymes, and epigenetic modifiers. A**. NO inhibits key metabolic enzymes in mitochondria, including components of the electron transport chain (ETC; complexes I, II, and IV), pyruvate dehydrogenase (PDH), oxoglutarate dehydrogenase (OGDH), and branched-chain α-ketoacid dehydrogenase (BCKDH), leading to metabolic reprogramming and reduced acetyl-CoA availability for histone acetylation. **B**. NO directly targets 4Fe-4S cluster-containing enzymes (e.g., aconitase 1/IRP1 and aconitase 2), disrupting iron-sulfur clusters and promoting formation of dinitrosyl iron complexes (DNICs), which lead to loss of activity. This also indirectly affects acetyl-CoA levels. DNIC formation can also occur through coordination of NO with iron centers ligated by histidine or aspartate residues in non-Fe-S proteins. **C**. These redox-dependent modifications influence iron-dependent dioxygenases, including KDM histone demethylases, TET DNA demethylases, FTO and ALKBH5 RNA demethylases, and prolyl hydroxylases (PHDs), thereby impacting chromatin and epigenetic regulation. **D**. In parallel, NO mediates protein S-nitrosation of cysteine residues and can affect zinc-binding domains through cysteine modification, altering protein structure and function. This is the case of NO-mediated modulation of sirtuin family deacetylases (SIRT1, SIRT2, SIRT6), further linking redox signaling to transcriptional control (e.g., histone acetylation and methylation).

The NO-induced inhibition of Fe-S proteins has direct metabolic consequences that contribute to the Warburg effect. The cellular control of iron is influenced by different variables, including NO. Iron regulatory proteins (IRPs) contain a Fe_4_S_4_ iron cluster that exhibits aconitase activity, along with a labile iron citrate at the apical location [115]. The occupation of the site by holoproteins signifies adequate iron levels, which then stimulate the expression of Ferritin (FTH1, FTL) for iron storage, while the transferrin receptor (TFRC) and iron regulatory proteins are downregulated. In the absence of iron at this location (apoprotein), genes such as TFRC, ferroportin, and aminolevulinate synthase 2 (ALAS2) can be indirectly regulated through NO-driven effects on iron-dependent metabolic enzymes [116]. Under normal conditions, mitochondrial acetyl-CoA serves as the primary substrate for histone acetyltransferases (HATs). However, when NO inhibits the TCA cycle through ACO2 inactivation and other Fe-S protein targets, the resulting decrease in mitochondrial acetyl-CoA synthesis leads to global histone hypoacetylation [117] (Fig. 5A–B).

### 7.2 Direct epigenetic targeting by nitric oxide

Beyond its indirect metabolic effects, NO directly targets multiple classes of epigenetic enzymes through its interaction with non-heme iron cofactors. The Fe(II)/2-oxoglutarate-dependent dioxygenase (2-ODD) family of enzymes, which includes histone demethylases (KDMs), DNA demethylases (TET enzymes), and RNA demethylases (fat mass and obesity-associated protein (FTO)), AlkB homolog 5 (ALKBH5)), all contain mononuclear non-heme iron atoms coordinated by the characteristic 2-His-1-carboxylate facial triad in their catalytic sites. NO binds directly to the iron atom in these active centers, forming dinitrosyl iron complexes that prevent oxygen binding and substrate catalysis. This mechanism represents the first demonstration of direct inhibition of histone demethylase activity by any endogenously produced small molecule. Inhibition by NO is reversible but persistent, with demethylases remaining inhibited as long as NO persists or DNICs remain at the catalytic site [[118], [119], [120]] (Fig. 5C).

TET enzymes represent a particularly important target in this context. These enzymes sequentially oxidize 5-methylcytosine (5 mC) to 5-hydroxymethylcytosine (5hmC), 5-formylcytosine (5 fC), and 5-carboxylcytosine (5caC), facilitating active DNA demethylation. NO inhibits TET enzymes with an IC_50_ of approximately 1 μM, preventing the conversion of 5 mC to its oxidized derivatives [119]. This inhibition results in genome-wide accumulation of both 5 mC and 5hmC at gene-regulatory regions, particularly at promoters and enhancers of cancer-associated genes. The coordinate inhibition of DNA, RNA, and histone demethylases establishes NO as a master regulator of cellular methylation dynamics, operating through a unified mechanism that targets the conserved iron-binding domain across all Fe(II)/2-OG-dependent enzymes [120].

Similarly, NO inhibits Jumonji C domain-containing histone demethylases such as lysine demethylase 3A (KDM3A), leading to increased methylation at key gene-regulatory histone residues, including H3K9me2, H3K27me2/me3, and H3K4me2/me3. Studies have shown that NO exposure results in significant alterations in numerous histone posttranslational modifications across multiple lysine residues on core histones H3 and H4. These methylation changes correlate with the differential expression of over 6500 genes in cancer cells, with transcriptional responses consistent with the induction of an oncogenic phenotype [121]. Notably, some modifications persist after NO removal (H3K27me2), while others return to baseline within 24 h (H3K9me2), suggesting that differential gene expression patterns can persist beyond the duration of NO exposure.

### 7.3 The prolyl hydroxylase (PHD) connection

NO regulates HIF-1α stability by directly inhibiting PHDs, which are 2-oxoglutarate–dependent dioxygenases (2-ODDs) that normally target HIF-1α for proteasomal degradation under normoxic conditions (Fig. 5C). Research demonstrated that HIF-1α accumulation occurs at intermediate NO concentrations (50-300 nM steady-state), representing a distinct threshold that is lower than that required for p53 activation (>300 nM) but higher than ERK phosphorylation (<50 nM) in MCF7 cells [122]. The PHDs contain a mononuclear non-heme iron atom coordinated by the characteristic 2-His-1-carboxylate facial triad in their catalytic sites, making them vulnerable to NO-mediated inhibition. Like the DNA and histone demethylases, NO binds directly to the iron center, forming DNICs that prevent oxygen binding and substrate catalysis, effectively blocking the hydroxylation of proline residues on HIF-1α that would normally signal for its degradation. This inhibition is reversible but persistent, with the enzyme remaining inhibited as long as NO is present or DNICs persist at the catalytic site. Importantly, the oxygen dependence of this mechanism creates a regulatory system in which hypoxic conditions enhance NO biological half-life and its ability to inhibit PHDs, thereby amplifying HIF-1α stabilization. The reaction between NO and superoxide can limit this signaling by reducing steady-state NO concentrations, as superoxide fluxes require higher NO concentrations to achieve the same level of HIF-1α accumulation [65], highlighting the critical balance between NO bioavailability and cellular oxygen consumption in regulating HIF-1α.

### 7.4 Sirtuin deacetylases and nitrosative stress

N-Myc downstream-regulated gene 1 (NDRG1), a metastasis suppressor protein, exemplifies the functional consequences of NO-iron interactions. NO upregulates NDRG1 expression through its interaction with the CIP, effectively sequestering iron in the form of DNICs and preventing the function of iron-dependent transcriptional repressors [[123], [124], [125], [126]]. The regulation of NDRG1 by NO provides another functional role for DNIC formation in gene expression. NDRG1 upregulation occurs independently of HIF-1α, N-Myc, c-Myc, and sGC signaling, but correlates directly with the DNIC to CIP ratio rather than absolute NO concentration. Functionally, NO-induced NDRG1 expression suppresses tumor cell migration, with iron supplementation completely abolishing this anti-metastatic effect. The magnitude of NDRG1 expression is inversely proportional to the amount of bioavailable chelatable iron, demonstrating that iron sequestration via DNIC formation is the primary mechanism driving transcriptional changes [123].

The relationship between NO and histone acetylation involves additional complexity through its effects on sirtuin deacetylases and classical heme protein interactions. Heme proteins represent the primary direct targets of NO, with sGC being the most sensitive biological target, requiring only nanomolar NO concentrations for its activation [[127], [128], [129]]. The chemistry of heme-NO interactions encompasses not only classical signaling through sGC but also regulatory effects on cytochrome *c* oxidase, catalase, and other heme-containing enzymes that control cellular metabolism and redox homeostasis. These NAD^+^-dependent sirtuin enzymes are sensitive to nitrosative modifications, with NO-derived oxidants inhibiting sirtuins (Sirt1, Sirt2, and Sirt6) through S-nitrosation of critical zinc-tetrathiolate cysteine residues, thereby reducing deacetylase activity and promoting histone hyperacetylation [130,131] (Fig. 5D). This dual mechanism, decreased acetyl-CoA availability due to metabolic reprogramming and direct inhibition of deacetylases, creates a complex regulatory network governing histone acetylation states [132].

### 7.5 Pathological implications and genomic instability

The inhibition of critical epigenetic modifications in NO-rich microenvironments creates a state of "epigenetic lock," in which normal gene expression programs cannot be properly regulated by physiological stimuli, such as those mediated by interferon gamma (IFN-γ), tumor necrosis factor alpha (TNF-α), or IL-1β. Beyond gene expression changes, NO-mediated iron sequestration provides cytoprotective effects against oxidative stress [119,133,134]. Cancer cells exposed to NO show significant protection against hydrogen peroxide-mediated cytotoxicity through iron sequestration in DNICs, which prevents iron-catalyzed Fenton chemistry and reduces reactive oxygen species (ROS) formation. This antioxidant function of NO may provide a selective survival advantage for NO-producing tumors against host defense mechanisms, potentially explaining why NO-associated cancers correlate with increased aggressiveness and worse patient outcomes [135]. This dual role, epigenetic dysregulation coupled with cytoprotection, may contribute to DNA damage tolerance and genomic instability, characteristic features of cancer progression. Under normal circumstances, such damage would activate p53-dependent DNA repair pathways, potentially leading to the suppression of NOS2 and COX2 expression via negative feedback. However, in the context of mutant p53, this protective response is compromised, amplifying carcinogenic signaling and promoting tumor progression.

The relationship between NO and iron complexes represents a fundamental regulatory axis that integrates metabolism, epigenetics, and gene expression. Through its effects on Fe-S proteins, NO induces Warburg-like metabolic reprogramming that indirectly alters histone modifications by altering acetyl-CoA availability. Simultaneously, NO directly targets iron-containing epigenetic enzymes, creating a coordinated methylation signature across DNA, RNA, and histones that favors oncogenic gene expression patterns [133].

This dual mechanism, metabolic reprogramming coupled with direct epigenetic targeting, provides a molecular framework for understanding how NO contributes to cancer progression beyond its traditional roles in inflammation and vascular biology. The oxygen dependence of NO-mediated signaling adds another layer of complexity, as different NOS isoforms (eNOS, iNOS, nNOS) produce NO with distinct spatiotemporal dynamics and oxygen sensitivities, creating unique epigenetic signatures in different tissue contexts [136,137]. Under hypoxic conditions, the biological lifetime of NO is extended, enhancing its ability to form DNICs and inhibit epigenetic enzymes. The convergence of NO, iron, and hypoxia on fundamental processes of cellular metabolism and gene regulation establishes NO as a central coordinator of the TME. Therapeutic strategies targeting this NO-iron-epigenetic axis may benefit from consideration of tissue oxygen levels and NOS isoform expression patterns to effectively reverse pathological gene expression programs and restore normal cellular homeostasis [137].

The concept of NO as an endogenous epigenetic regulator, rather than merely a classical second messenger, represents a paradigm shift in our understanding of both NO signaling and epigenetic control mechanisms. This expanded view of NO as a central coordinator of the TME provides novel therapeutic targets for conditions characterized by dysregulated NO production and aberrant epigenetic landscapes.

## 8 Regulatory factors of NO flux

The production and consumption of NO are critically flux-dependent. Optimal induction of NOS2 in human tumor cells occurs when IFNγ is combined with TNF-α and/or IL-1β, similar to conditions used to induce Nos2 in murine models. RNA-seq analysis has shown that high NOS2 expression in human tumors is associated with microenvironments enriched in these Th1 cytokines, indicating that human NOS2, like murine Nos2, requires robust Th1-type inflammatory signaling for full induction [138].

Despite these similarities, murine and human NO levels in vitro differ, in part because of differences in the frequency of cells expressing NOS2 and in the architecture of the NOS2 promoter [138,139]. At the protein and enzymatic levels, however, the mouse and human NOS2 exhibit substantial conservation. The two proteins share approximately 80% sequence identity based on pairwise genomic and protein alignments [140] and display nearly identical enzymatic properties, including similar Km values for arginine and oxygen [141,142]. These molecular and biochemical similarities indicate that species-specific differences in NOS2 function primarily arise from differences in expression and regulation rather than from intrinsic catalytic properties [143].

Comparative analysis of the promoter regions underscores this regulatory divergence. The murine Nos2 promoter contains canonical GAS (IFNγ-responsive), NF-κB, and AP-1 elements that drive rapid induction by IFNγ, Th1 cytokines, and LPS [144]. The human NOS2 promoter also harbors AP-1, STAT, and NF-κB sites, as well as putative GAS motifs recognized by activated signal transducer and activator of transcription (STAT) factors (particularly STAT1) following IFNγ stimulation [[145], [146], [147], [148]]. However, these GAS motifs are positioned more distally, and their functional impact appears less direct than in the murine promoter. In addition, the human NOS2 promoter contains a β-catenin/TCF4 (LEF/TCF) binding element which is absent in the mouse Nos2 promoter, providing an additional layer of regulation linked to Wnt/β-catenin signaling [149].

Hypoxia further distinguishes the regulation of murine and human NOS2. In mice, defined hypoxia-responsive elements within the Nos2 promoter integrate low-oxygen and metabolic cues, such as iron depletion and ascorbate status, aligning Nos2 expression with HIF-dependent pathways. Non-heme iron PHDs, which regulate HIF-1α in an oxygen-dependent manner, also modulate NF-κB signaling via effects on IKKβ. Specifically, PHD1-mediated hydroxylation can negatively regulate IKKβ, whereas hypoxia decreases IKKβ hydroxylation, thereby activating IKKβ and NF-κB signaling [150]. Together, these observations support the conclusion that the principal differences between murine and human NOS2 lie in transcriptional and contextual regulation, driven by promoter architecture, signaling inputs, and microenvironmental conditions, rather than in the underlying molecular or biochemical properties of the NOS2 enzyme itself.

### 8.1 The consumption of NO and regulation of pO_2_ gradients

NO flux is determined not only by its production but also by its consumption. Although the mechanisms driving NOS2 induction are well characterized, NO uptake and removal are equally important in shaping its flux. Both NO synthesis by NOS and its oxidative consumption are oxygen-dependent processes, and they exhibit similar apparent Km values for O_2_ in murine macrophages [136]. Experimental mapping in these cells indicates that optimal NO flux occurs at intermediate oxygen tensions (approximately 2-5% O_2_), closely matching physiological tissue oxygen levels [136,151]. Interestingly, comparable net NO flux is observed at 1% and 20% O_2_, suggesting that under hypoxia, NO production declines while NO half-life increases, resulting in similar effective NO levels despite different oxygen tensions [136].

Importantly, NO bioavailability and half-life are not determined solely by oxygen tension and ROS flux, but also by the relative abundance of intracellular NO reservoir systems generated by low-molecular-weight thiols such as glutathione and acetyl-CoA. The formation and turnover of nitrosated intermediates, including S-nitrosoglutathione (GSNO) and S-nitroso-CoA, together with the activity of denitrosylases, can substantially modulate NO persistence, spatial diffusion, and downstream nitrosative signaling within the TME [136].

Because mitochondrial respiration is the principal sink for O_2_, NO-mediated inhibition of mitochondrial ETC reduces local O_2_ consumption and steepens the pO_2_ gradient across tissues [151]. Near the hypoxic threshold, NO generated in better-oxygenated regions can diffuse into adjacent hypoxic zones, where OXPHOS inhibition further limits O_2_ utilization, effectively allowing more O_2_ to penetrate deeper into the tissue. This NO-dependent redistribution of O_2_ selectively increases diffusion into hypoxic tumor regions, thereby reshaping the TME without necessarily reversing the Warburg-like metabolic shift in cancer cells [151].

## 9 Nitric oxide-driven cyclic nucleotide signaling and metabolic zonation in the tumor microenvironment

**Fig. 6:**
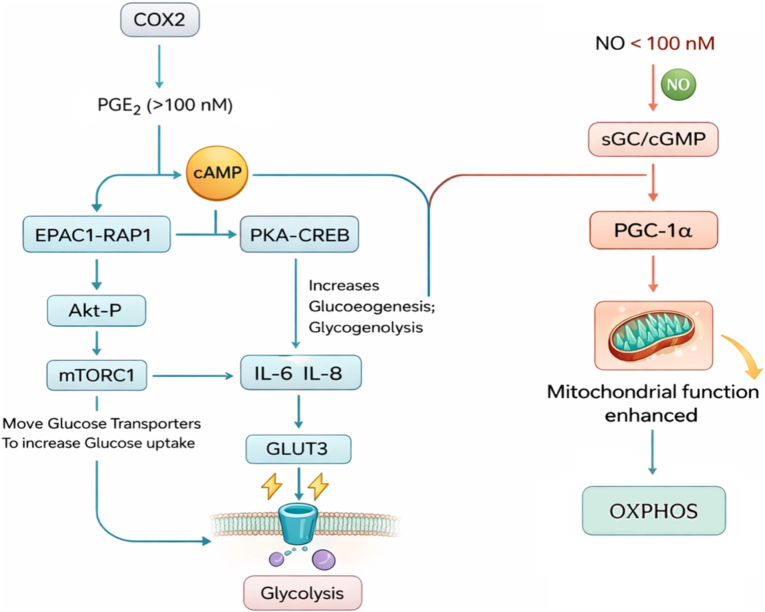
**Divergent metabolic programming driven by COX2-cAMP and low-flux NO-cGMP signaling within the tumor microenvironment. Left panel:** COX2-derived prostaglandin E_2_ (PGE_2_; >100 nM) elevates intracellular cAMP through EP receptor activation, initiating parallel EPAC1-RAP1 and PKA-CREB signaling cascades. EPAC1-mediated RAP1 activation promotes Akt phosphorylation and mTORC1 engagement, enhancing GLUT trafficking to the plasma membrane and increasing glucose uptake. Concurrently, PKA-CREB signaling induces IL-6 and IL-8 expression, which act in autocrine and paracrine loops to further amplify glycolytic gene transcription and upregulate GLUT3. These coordinated cAMP-dependent events reinforce aerobic glycolysis, support anabolic flux, and stabilize a Warburg phenotype within COX2-enriched tumor niches. **Right panel:** At lower nitric oxide concentrations (<100 nM), NO preferentially activates soluble guanylate cyclase (sGC), increasing cGMP and stimulating downstream PKG-dependent signaling that induces PGC-1α. PGC-1α enhances mitochondrial biogenesis, promotes oxidative phosphorylation (OXPHOS), and counterbalances glycolytic commitment, thereby establishing an “anti-Warburg” metabolic state.

The interplay of COX2, cyclic adenosine monophosphate (cAMP), NO, and cGMP can differentially affect cancer cell metabolism. Increased NOS2 and COX2 in the TME can modify cancer cell metabolism both through direct chemical interactions and through cAMP and cGMP signaling, which differentially influence mitochondrial status. This section illustrates that nitrosative signaling, together with COX2/PGE_2_/cAMP, can collaboratively enhance a Warburg or induce an anti-Warburg metabolic shift, thereby regulating mitogenesis and mitophagy (Fig. 6) [152].

### 9.1 NOS2 and COX2 in distinct but cooperative tumor niches drive context-dependent Warburg and anti-Warburg reprogramming

The presence of NOS2 and COX2 in separate, but spatially related, cell populations implies the formation of discrete cellular microenvironments within the tumor that mutually enhance their expression while synergistically modifying cancer cell metabolism. As discussed above, co-expression of NOS2 and COX2 in malignancies is associated with poor clinical outcomes. Local NOS2 generates NO, which reduces mitochondrial activity and increases glycolysis and lactate production, effects that are further augmented by COX2-mediated cAMP synthesis.

In contrast, NO/cGMP can increase mitogenesis by activating protein kinase G (PKG), which upregulates master regulators such as peroxisome proliferator-activated receptor gamma coactivator 1-alpha (PGC-1α), nuclear respiratory factor 1 (NRF-1), and mitochondrial transcription factor A (TFAM), thereby increasing mitochondrial biogenesis, improving cellular energy production, and countering mitochondrial dysfunction. At low doses of NO, cGMP signaling can significantly impact the Warburg effect by promoting a shift away from aerobic glycolysis toward OXPHOS. This effect is often described as an “anti-Warburg effect” [[152], [153], [154]]. Areas with low oxygen concentrations or located at a distance from NOS2 clusters tend to favor NO/cGMP signaling, which is characteristic of this “anti-Warburg effect”(152). The activation of PGC-1α enhances mitochondrial function and reduces reliance on glucose for energy [155]. Increased cGMP, often via NO/cGMP/PKG pathways, can induce differentiation and suppress cancer cell growth by reprogramming metabolism, in direct contrast to the Warburg phenotype (Fig. 6). Thus, tumor cells far from NOS2-positive cells would be expected to increase OXPHOS and undergo an “anti-Warburg shift” [152].

PGE_2_ influences cancer metabolism through cAMP-linked receptors, oncogenic signaling, and interactions with immune and stromal cells [156] (Fig. 6). The Warburg effect can be augmented by cAMP in several cancers; however, the outcome is highly dependent on the specific pathway and cell type [157]. Promotion of aerobic glycolysis occurs primarily through exchange protein directly activated by cAMP 1(EPAC1)-Ras-related protein Rap-1 (RAP1) and protein kinase A (PKA)-cAMP response element-binding protein (CREB) signaling pathways, which connect glucose uptake and growth factor signals to glycolytic gene expression and integrin/AKT activation. In tumor cells and stromal fibroblasts, cAMP effectors, cAMP-PKA and cAMP-EPAC, can directly enhance aerobic glycolysis in cancer cells [158]. Consequently, NOS2 levels that facilitate COX2 expression in other cells also support the Warburg shift in NOS2-expressing cells. Regions exhibiting clustering of NOS2 and COX2 thus experience both direct chemical modification of mitochondria by NO and elevated cAMP, fostering an environment that enhances the Warburg effect and promotes metastasis and other malignant traits [34]. NOS2 and COX2 are frequently observed in niches but typically reside in different cells, forming distinct “neighborhoods” that reinforce their expression. Steady NO flux triggers PGE_2_ synthesis, elevating cAMP levels (Fig. 6). cAMP signaling has a variety of pro-tumor effects, including enhanced angiogenesis and increased IL-6 production. More broadly, cAMP influences cancer metabolism by enhancing aerobic glycolysis and fatty acid oxidation, potentially conferring growth and survival advantages while also creating metabolic vulnerabilities [13,158,159].

In other contexts, cAMP and PGE_2_ can contribute to an anti-Warburg effect by activating pathways that shift metabolism toward OXPHOS and away from glycolysis, often promoting cellular differentiation in cancer or specific immune cell functions [152]. Similar to cGMP, increased intracellular cAMP can activate PKA, which phosphorylates CREB and induces PGC1α expression [160] (Fig. 6). In addition, many cancers that produce high levels of PGE_2_ and adenosine promote immune suppression [152,161]. Overall, the impact of NO/cGMP and PGE_2_/cAMP is concentration-, spatial-, and temporal-dependent and is expected to be a major determinant of TME organization and metabolic status.

### 9.2 Nitric oxide and the reverse Warburg effect

**Fig. 7:**
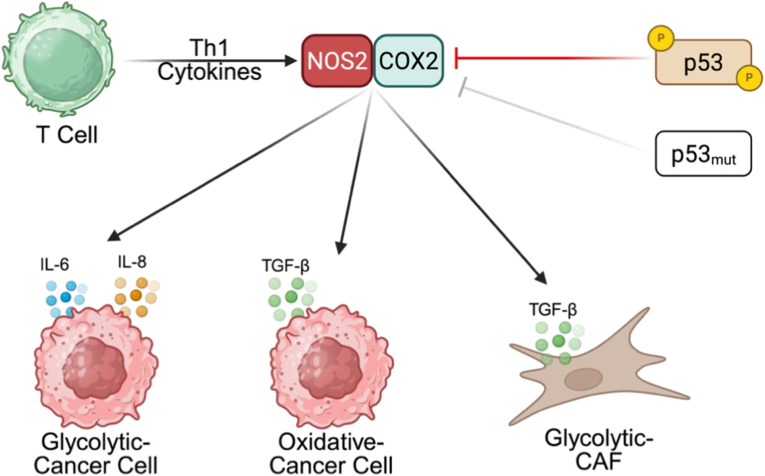
**Immune-driven NOS2/COX2 activation and cytokine-mediated metabolic reprogramming in the tumor microenvironment.** Th1 cytokines released by activated T cells induce NOS2 and COX2 expression, leading to increased production of IL-6, IL-8, and TGF-β. These cytokines then act on cancer cells and stromal cells, including glycolytic cancer cells, oxidative cancer cells, and glycolytic cancer-associated fibroblasts (CAFs), to promote metabolic reprogramming toward protumor phenotypes. Activated p53 negatively regulates NOS2/COX2, thereby restraining cytokine production and downstream metabolic changes, whereas mutant p53 fails to inhibit NOS2/COX2, allowing sustained inflammatory signaling and maintenance of tumor-supportive metabolic states.

**Fig. 8:**
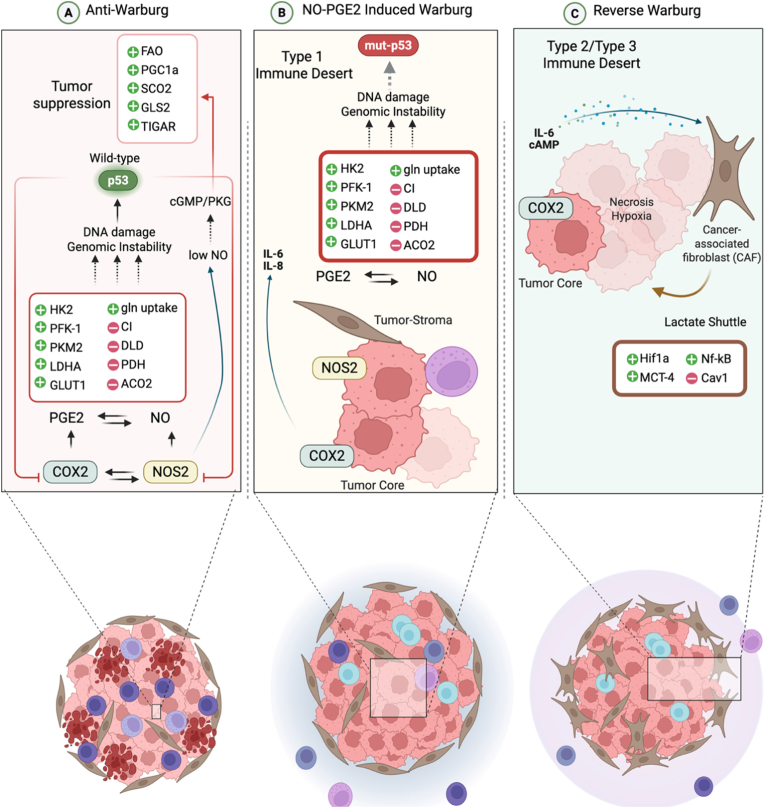
**Stage-specific coupling of the Warburg effect with NOS2 and COX2 signaling drives immune desert phenotypes**. **A.** In tumors retaining wild-type p53, DNA damage-induced p53 activation suppresses the Warburg effect by restraining glycolytic enzymes and glutamine uptake while promoting mitochondrial respiration. In this oxidative state, COX2 and NOS2 expression remain low and tightly regulated, with balanced PGE_2_ and NO production. NOS2-derived NO levels are limited, preserving cGMP/PKG signaling and preventing feed-forward glycolytic amplification. This supports immune surveillance and prevents the establishment of an immune desert. **B.** Loss of p53 function leads to sustained tumor NOS2 and COX2 expression. This enhances aerobic glycolysis and glutamine metabolism with reduced mitochondrial oxidative capacity. The resulting lactate-rich, redox-altered tumor core establishes a metabolically inflamed but immune-excluded niche. NOS2-derived NO and COX2-derived PGE2 form a feed-forward loop that reinforces glycolysis, promotes IL-6 and IL-8 production, and sustains tumor–stroma signaling. In this stage, NOS2 at the tumor-stroma interface and COX2 in the tumor core coordinate metabolic reprogramming and immune exclusion characteristic of a glycolysis-driven Type 1 immune desert. **C.** Persistent Warburg metabolism promotes hypoxia and necrosis within the tumor core, stabilizing HIF-1α and activating NF-κB. In these advanced stages, COX2 expression becomes prominent within the hypoxic tumor core, while stromal elements, including cancer-associated fibroblasts (CAFs), are activated by cytokines produced by COX2+ tumor cells, and contribute to inflammatory signaling and metabolic coupling. HIF-1α-driven monocarboxylate transporter 4 (MCT-4) upregulation supports lactate export, enabling lactate shuttling between tumor cells and CAFs. This axis amplifies PGE2 and NO signaling, reinforces hypoxia-adapted glycolysis, and consolidates immune exclusion in Type 2/3 immune deserts.

In the reverse Warburg effect, cancer-associated fibroblasts (CAFs) and other stromal cells are pushed into a state of aerobic glycolysis, exporting lactate, pyruvate, and related metabolites that fuel OXPHOS in neighboring, more oxidative tumor cells [162] (Fig. 7, Fig. 8). NO is a key trigger of this metabolic compartmentalization: increased NO production in fibroblasts, such as following caveolin-1 loss, impairs mitochondrial function, enhances ROS levels, and stabilizes HIF-1α and NF-κB, which together drive glycolytic enzyme expression, autophagy, and mitophagy, locking these stromal cells into a glycolytic, catabolic phenotype that secretes high-energy metabolites to the tumor compartment [[162], [163], [164]]. In immune cells, NOS2-derived NO similarly inhibits mitochondrial enzymes and promotes a highly glycolytic, pro-inflammatory program, increasing local lactate and metabolite release. Tumor cells and immunosuppressive subsets can then oxidize these metabolites in their own TCA cycle and OXPHOS, creating an NO-linked division of labor where glycolytic, NO-stressed stromal and immune cells support the bioenergetic and biosynthetic demands of more oxidative cancer cells and contribute to immune evasion [20,162].

### 9.3 IL-6, IL-8, and cytokine-driven metabolic remodeling

IL-6 and IL-8 are key downstream cytokines induced by nitrosative signaling and the COX-PGE_2_ axis [36]. In breast cancer, COX2 induced by NOS2 leads to IL-6 production. IL-6 is a potent regulator of cancer cell metabolism, promoting glycolysis (Fig. 7) and, in some settings, altering mitochondrial function to support growth, survival, and metastasis. In colorectal cancer cell lines, IL-6 increases glucose consumption, lactate output, and expression of key glycolytic genes. Knockdown of PFKFB3 reverses these effects and limits proliferation and migration. IL-6 also shapes stromal cell metabolism, indirectly modulating cancer cell energetics [165]. In cancer-associated fibroblasts, IL-6 participates in a signaling network that drives a glycolytic, lactate-producing “reverse Warburg effect,” supplying high-energy metabolites to tumor cells, as discussed above. Together, these findings demonstrate that NO levels drive COX2 expression and increase cAMP, which can further support the Warburg effect and lipid utilization. Because COX2 is expressed in different cells, this implies that cAMP may regionally support the Warburg phenotype (mediated through NO) in some compartments of the TME (Fig. 6), while increasing lipid consumption in others.

IL-8, produced downstream of NOS2 induction and NO donors, promotes a more glycolytic, Warburg-like phenotype (Fig. 7) while supporting redox balance, thereby enhancing proliferation, invasion, and survival [166,167]. IL-8 can increase aerobic glycolysis in colorectal cancer cells by enhancing glucose uptake and lactate production, while lowering intracellular ROS, thereby supporting proliferation and invasion. Additionally, in pancreatic ductal adenocarcinoma, IL-8 produced by TAMs activates STAT3 in tumor cells, upregulates GLUT3, and drives increased glycolytic flux [168,169]. IL-6 and IL-8 also promote catabolic processes, such as lipolysis and glycolysis, contributing to systemic and local metabolic remodeling in the TME [170]. By enhancing glycolysis and reducing ROS, IL-8 signaling supports survival under metabolic stress and may facilitate adaptation to hypoxia and nutrient competition in the TME. Additionally, IL-6 and IL-8 from tumor and stromal cells (e.g., adipocytes, macrophages) promote tumor growth, EMT, and angiogenesis, creating a microenvironment that favors high glycolytic activity and nutrient delivery to cancer cells [[171], [172], [173]].

### 9.4 Spatial gradients of NO and cyclic nucleotides

The above-mentioned observations suggest that, starting from a point source of NOS2/NO, different NO gradients will exert distinct effects through cAMP or cGMP. In areas adjacent to NOS2^+^ cells, several mechanisms diminish cGMP signaling, including S-nitrosation and oxidation of the sGC- heme and sustained elevation of thrombospondin-1 (TSP1), a potent antagonist of cGMP signaling that also increases TGFβ activation [[174], [175], [176]]. At lower NO fluxes away from the source, such as under hypoxia or in regions distal to NOS2 clusters, the Warburg effect is expected to be reversed, with OXPHOS metabolism favored. From a spatial perspective, this would generate distinct metabolic clusters determined by NOS2 spatial localization and hypoxic regions. Taken together, these observations indicate that cyclic nucleotides, integrated with spatial NO gradients, can dramatically influence the “geography” of metabolic profiles within the TME.

## 10 The NOS2-COX2-p53 regulatory axis in advanced cancers

These aforementioned metabolic consequences of sustained NOS2 activity not only shape tumor bioenergetics but also converge with COX2 signaling and p53 regulation, forming an interconnected axis that links inflammatory cues to metabolic plasticity and genomic instability in advanced malignancies. As previously mentioned, under normal conditions, p53 mitigates chronic inflammatory signaling and metabolic stress by limiting the induction of NOS2 and COX2, keeping NO and PGE_2_ production within a range compatible with tissue homeostasis (Fig. 7) [177,178]. In many aggressive tumors, however, p53 is lost or mutated [99], while NOS2 and COX2 become persistently upregulated in cancer cells and tumor-associated myeloid populations, creating a self-reinforcing loop of inflammatory signaling, redox imbalance, and bioenergetic rewiring. This deregulated triad amplifies NO- and PGE_2_-driven effects on glycolysis, mitochondrial function, and DNA damage responses, promoting the survival and adaptation of genetically unstable clones and positioning the NOS2-COX2-p53 axis as a central hub through which advanced cancers couple inflammatory cues to metabolic plasticity, immune evasion, and therapy resistance [13,14,99].

The feedforward relationship between NOS2 and COX2 is crucial not only for the expression of each enzyme but also for their collective impact on the TME. In this context, sustained NOS2/COX2 activation elevates IL-6 and IL-8 levels within the TME, which act in a paracrine and autocrine manner to enhance glucose uptake, glycolytic enzyme expression, and lactate production in tumor cells, thereby reinforcing and stabilizing a Warburg-like metabolic phenotype (Fig. 7). Elevation of PGE_2_ and IL-6 sustains NOS2 and COX2 expression, whereas increases in TNF and IL-1 further drive COX2 induction [14,30]. Both NO and PGE_2_ play complementary roles in reshaping the tumor niche, promoting metastasis, chemoresistance, cancer stemness, and immune suppression [13]. In normal and immune cells, high NOS2/NO levels can eventually stabilize p53, which then suppresses NOS2 and COX2 transcription [179].

Increased expression of NOS2 and COX2 enzymes has been reported in breast, liver, gastric, esophageal, colorectal, glioma, prostate, lung, ovarian, cervical, and head-and-neck cancers [13]. Mechanistically, stromal lymphoid aggregates produce IFNγ and Th1 cytokines that induce NOS2 and COX2 (Fig. 7). These enzymes promote immunosuppression, leading to a gradual decrease in immune activity and ultimately reducing NOS2 and COX2 levels as the TME becomes less inflamed [34,36,107]. In p53-mutant tumors, the presence of COX2 may therefore mark prior NOS2 activity, while chronic NO exposure itself can promote p53 and PI3K mutations, as observed in metaplastic breast cancers with very high NOS2 levels [180,181]. Taken together, these observations support a model in which p53-mutated, COX2-high tumors exhibit NO peaks driven by NOS2-mediated inflammation, which fuel metastasis and immunosuppression.

## 11 Integrated direct and indirect nitric oxide mechanisms in tumor metabolic reprogramming

The spatial metabolic zonation created by NO gradients, cyclic nucleotide signaling, and the COX2/PGE_2_ axis is further stabilized by the tight coupling of direct biochemical effects of NO on metabolic enzymes with its indirect effects on transcriptional and signaling networks. Together, these NO-driven effects generate self-reinforcing metabolic programs that keep cells in relatively stable, therapy-resistant states. In NO-producing TAMs and cancer cells, direct inhibition of ACO2 and PDH increases glutamine uptake and utilization, generating α-KG that partially restores TCA cycle function and supports biosynthetic reactions [50]. This adaptation comes at a cost: NO-reprogrammed cancer cells become glutamine-addicted and, by consuming large amounts of glutamine, deplete this critical nutrient from the TME, thereby impairing T cell proliferation, effector function, and redox balance in neighboring immune cells. In parallel, NO indirectly activates important regulatory pathways such as Akt/mTOR and HIF-1α, further enhancing glutamine uptake and metabolism, aligning signaling outputs with the new metabolic demands. A similar logic applies to glucose: suppression of OXPHOS forces higher glycolytic flux to maintain ATP levels, and NO-responsive signaling increases glucose transporter and glycolytic enzyme expression to meet this substrate demand, while depleting glucose and acidifying the TME with lactate, thereby suppressing immune function [182].

The metabolic rewiring promoted by NO exposure, through substrate depletion, accumulation of reactive intermediates, and redox imbalance, is buffered by indirect coordinated transcriptional and metabolic programs that enhance glutamine anaplerosis, activate antioxidant systems (e.g., Nrf2 and glutathione pathways), and engage alternative bioenergetic circuits that preserve cellular viability. As a result, cells do not readily revert from an NO-conditioned state even when NO levels decline, because both the metabolic machinery and the signaling architecture have shifted to a new homeostatic set point. Within the TME, this generates a stable scenario in which NO-producing cells remodel the metabolic landscape both by directly acting on neighboring cells and by indirectly altering shared metabolite pools, a process we refer to as “tumor interstitial fluid (TIF)-mediated trans effects.” Metabolite changes in the TIF, including arginine depletion, citrulline accumulation, citrate elevation, lactate accumulation, glutamine depletion, and altered redox status, with different independent mechanisms converging to impair immune cell function [[183], [184], [185], [186]].

p53 status adds a further layer of control over how cells respond to NO. Activation of p53 marks a critical decision point, with outcomes determined by the balance between repair and damage, p53 mutational status, and concurrent survival signals. When intact, p53 can repress NOS2, allowing cells to recover if NO exposure is transient. Under chronic inflammation or sustained tumor-associated NO production, however, this circuitry can be overridden or bypassed. Beyond classical cell-fate control, p53 directly reshapes metabolism: it can inhibit mitophagy, limiting clearance of damaged mitochondria and potentially worsening mitochondrial dysfunction, while simultaneously promoting β-oxidation through upregulation of fatty acid oxidation enzymes and enhancement of CPT1 activity, shifting metabolism away from glycolysis toward oxidative metabolism in an attempt to restore mitochondrial function [187]. These p53-driven metabolic changes create cellular states distinct from those induced by lower NO levels, with important consequences for both tumor and immune cells depending on p53 status and the broader mutational landscape. When p53 is mutated, its impact on metabolism can synergize with NO-mediated changes. Mutant p53 can suppress OXPHOS, enhance glycolysis, and promote the Warburg effect by upregulating GLUTs and glycolytic enzymes and by impairing mitochondrial function, so that genetic and metabolic alterations converge to drive more aggressive cancer behavior [188,189] (Fig. 8).

Finally, the spatial organization and density of NOS2-expressing cells within the TME determine the reach and magnitude of these direct and indirect mechanisms, creating distinct metabolic niches that can now be visualized with emerging spatial metabolomics and imaging approaches. In regions with high NOS2 expression and dense cellular clustering, NO flux can exceed ∼500 nM, causing nitrosative stress, irreversible mitochondrial damage, and potential cytotoxicity to both cancer and immune cells. Such microenvironments may undergo necrosis, contributing to hypoxic, acidic tumor cores, with necrotic debris releasing damage-associated molecular patterns (DAMPs) that paradoxically fuel tumor growth via inflammatory signaling. At the margins of these NOS2-rich areas, intermediate NO levels (200-500 nM) favor nitrosative signaling rather than cytotoxicity, promoting cancer stem-like features, metastatic potential, and immune suppression without inducing cell death, consistent with the aggressive, therapy-resistant phenotypes often seen at tumor edges. In more distant regions, or where NOS2 density is lower, NO levels may fall into the cGMP-signaling range (<100 nM), where effects on sGC, neurogenic locus notch homolog protein (NOTCH), and related pathways predominate without major metabolic disruption, supporting angiogenesis and tumor cell survival [13,35,190]. This spatial heterogeneity creates a metabolic mosaic within a single tumor, with region-specific vulnerabilities and therapeutic responses, and with TIF composition varying across niches to provide locally optimized conditions for tumor expansion and immune evasion.

## 12 NOS2-COX2 niches and the spatial-metabolic architecture of immune deserts in ER- breast cancer

**Fig. 9:**
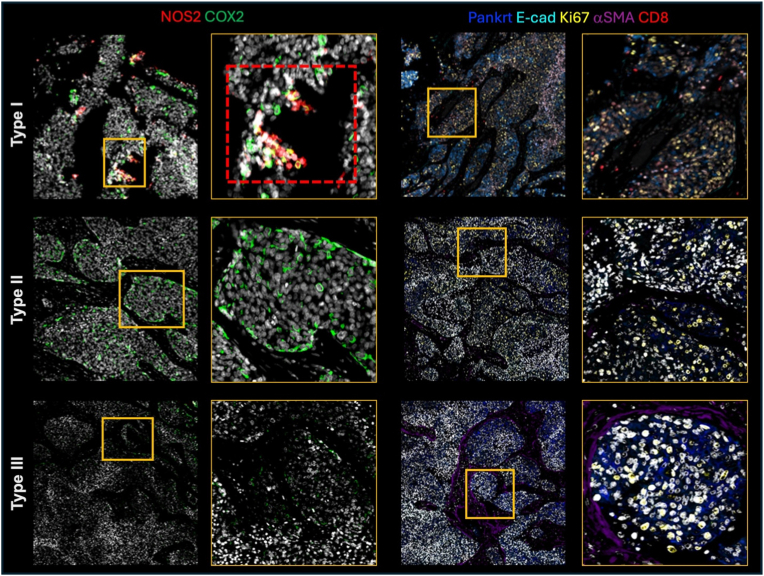
**Type I-III immune deserts in estrogen receptor-negative breast cancer stained for inflammatory, epithelial, proliferative, stromal, and T-cell markers. Left Panel -** Multiplex immunofluorescence for NOS2 (red), COX2 (green), and nuclei (gray) illustrates NOS2-COX2 niches across Type I-III immune deserts. Type I regions show prominent NOS2 clusters with adjacent COX2-positive tumor and stromal cells at the tumor-stroma interface, whereas Type II areas go deeper in the tumor and display reduced NOS2 with sustained tumor COX2 expression, and Type III lesions are largely NOS2-low with scattered COX2 positivity at the periphery of necrotic cores. **Right panel -** Parallel sections stained for pan-keratin (Pankrt, cyan), E-cadherin (E-cad, green), Ki67 (yellow), alpha smooth muscle actin (αSMA) (magenta), CD8 (red), and nuclei (blue) define tumor epithelium, proliferative index, cancer-associated fibroblasts, and cytotoxic T cells within each immune-desert type. Type I fields retain stromal CD8^+^ T cells around NOS2 clusters, Type II regions show loss of CD8^+^ cells with αSMA-rich myeloid and fibroblast stroma, and Type III mature deserts exhibit αSMA-positive fibroblast rims encasing highly proliferative, hypoxic tumor islands with minimal lymphoid infiltrate.

A key step in understanding how NO and COX2 shape tumor behavior is to consider their spatial organization, since NOS2/COX2 niches promote the establishment of distinct types of immune deserts. In ER^−^ breast cancer, clusters of tumor-associated NOS2 appear in defined regions that correlate with increased metastatic risk [34,36,107]. COX2 shows a similar pattern, forming clusters in more aggressive cancers, such as ER^−^ breast cancer. On this basis, three immune-desert configurations have been described in ER^−^ breast cacner: Type I “confined inflamed,” Type II “developing immune desert,” and Type III “mature immune desert” [34]. In Type I immune deserts, CD8^+^ T cells are present in the stroma and extratumoral regions, where they provide cytokines that organize lymphoid structures and induce NOS2, but they fail to penetrate the tumor core; here, NOS2 clusters in both tumor and stromal cells are associated with increased metastatic potential. Type II immune deserts show a sharp decline in extratumoral CD8^+^ T cells and a predominance of macrophages and other myeloid cells; NOS2 is low, whereas tumor COX2 is high in these regions. Type III immune deserts are characterized by profoundly reduced leukocytes and a frequently hypoxic core, representing a later stage in a temporal sequence of cyclic inflammation that progressively drives tumor malignancy (Fig. 9).

Building on this spatial framework, the metabolic programs within each immune desert help drive disease progression across stages of ER^−^ breast cancer. In Type I immune deserts, NOS2 clusters localize to the tumor-stroma interface, while COX2 is more concentrated in the tumor core [107]. These regions are enriched for NO and PGE_2_ and are predicted to favor a Warburg effect, with elevated IL-6 and IL-8 from NOS2- and COX2-expressing cells further reinforcing aerobic glycolysis and tumor outgrowth. This combination of metabolic reprogramming and oncogenic signaling creates a highly permissive niche for metastasis. As the microenvironment becomes more immunosuppressive and transitions to a Type II immune desert, COX2 activity becomes dominant while NOS2 expression declines [34]. Under these conditions, IL-6 and cAMP-linked pathways can maintain Warburg-like metabolism despite reduced NOS2. In Type III mature immune deserts, COX2 expression declines as necrosis expands and hypoxia becomes a defining feature of the tumor core. In these regions, CAFs often form a rim around tumor nodules (Fig. 9), a pattern commonly associated with increased keratin 14 expression. Within this oxygen-poor, necrotic environment, a “reverse Warburg effect” emerges: CAFs shift toward glycolysis, releasing lactate and other energy-rich metabolites that neighboring tumor cells can use to sustain growth and survive under metabolic stress [191,192] (Fig. 1, Fig. 8).

## 13 Nitric oxide-driven Warburg reprogramming as an Integrative engine of cancer hallmarks

**Fig. 10:**
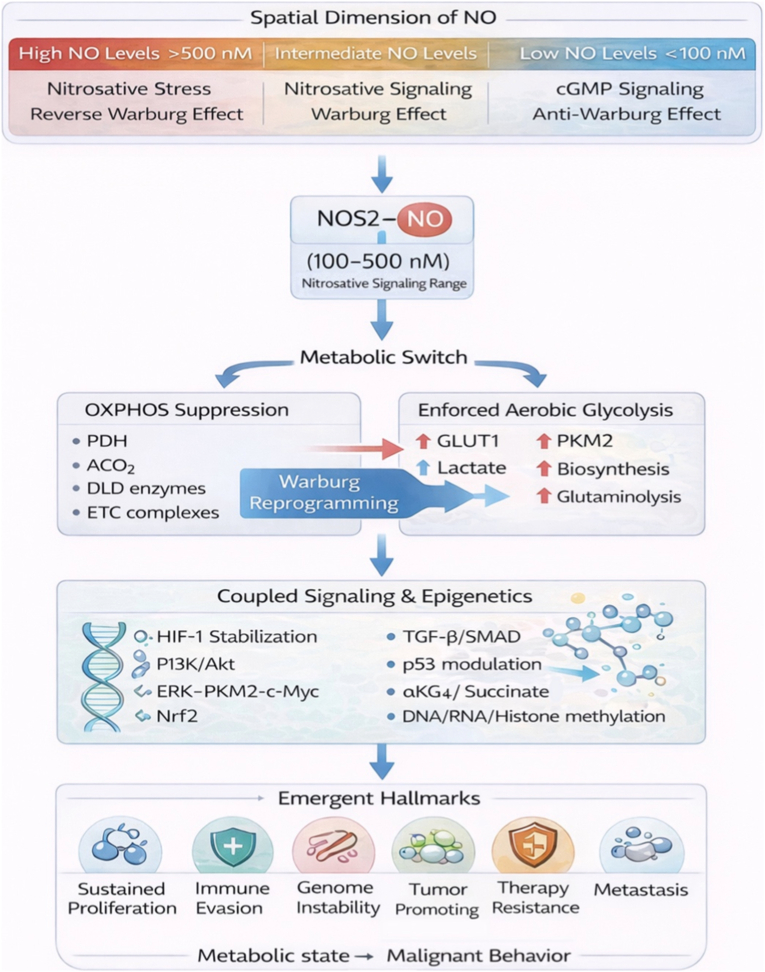
**Spatial and concentration-dependent regulation of tumor metabolism by NOS2-derived nitric oxide. The spatial dimension of NO establishes a hierarchical metabolic framework within the tumor microenvironment (TME).** High NO levels (>500 nM) promote nitrosative stress and reverse Warburg compartmentalization; intermediate levels (100-500 nM) sustain nitrosative signaling and enforce the Warburg phenotype; and low NO levels (<100 nM) preferentially activate sGC/cGMP signaling, supporting mitochondrial function and an anti-Warburg oxidative state. Within the nitrosative signaling range (≈100-500 nM), NOS2-derived NO acts as a metabolic switch that simultaneously suppresses oxidative phosphorylation (OXPHOS) and enforces aerobic glycolysis. Direct inhibition of mitochondrial targets, including pyruvate dehydrogenase (PDH), aconitase 2 (ACO_2_), DLD-containing dehydrogenase complexes, and electron transport chain (ETC) components, restricts TCA cycle flux and electron transport. This mitochondrial constraint drives Warburg reprogramming characterized by increased GLUT1 expression, PKM2 activation, lactate production, enhanced biosynthesis, and glutaminolysis. Concurrently, NO engages coupled signaling and epigenetic circuits, including HIF-1 stabilization, PI3K/Akt activation, ERK-PKM2-*c*-Myc signaling, Nrf2-mediated redox adaptation, TGF-β/SMAD signaling, and modulation of p53. Alterations in metabolite pools (α-ketoglutarate, succinate) and direct targeting of iron-dependent enzymes further influence DNA, RNA, and histone methylation, reinforcing metabolic commitment through epigenetic stabilization. These integrated metabolic, signaling, and epigenetic NO-driven effects promote different hallmarks of cancer, including sustained proliferation, immune evasion, genome instability, tumor-promoting inflammation, therapy resistance, and metastasis, linking metabolic state directly to malignant behavior.

NO-driven Warburg reprogramming offers a unifying perspective, viewing the hallmarks of cancer described by Hanahan as emergent features of a TME conditioned by NO. At nitrosative signaling levels in the range of roughly ≈100-500 nM, NOS2-derived NO simultaneously suppresses OXPHOS while enforcing aerobic glycolysis and activating a dense network of oncogenic signaling and epigenetic programs [35,41]. This concentration window indicates that NO is not merely a byproduct of inflammation but a central organizer that couples metabolic state to malignant behavior [13]. In Hanahan's work [193], this constitutes a highly evolved form of “deregulated cellular energetics” in which glucose is diverted toward lactate and biosynthesis, glutaminolysis sustains anaplerosis, and lipid handling shifts toward storage and selective consumption, all under the control of NOS2 activity and its spatially patterned expression in tumors (Fig. 10).

Within this NO-imposed metabolic architecture, several classical and emerging hallmarks of cancer converge. “Sustained proliferative signaling” and “replicative immortality” are reinforced as NO-stabilized HIF-1/Akt upregulate GLUT1 and a broad panel of glycolytic enzymes, ERK-driven PKM2 and c-Myc amplify transcription of glycolytic and anabolic genes, and Nrf2 and TGF-β supply antioxidant and biosynthetic capacity that allows high-flux glycolysis to persist despite redox stress [31,94]. The same Warburg program supports “resisting cell death” and “adapting to stress” by lowering mitochondrial ROS-induced apoptosis, modulating p53-dependent checkpoints, and, at higher fluxes, reshaping iron and lipid metabolism in ways that can promote ferroptotic vulnerability yet ultimately select for clones that tolerate hypoxia, acid, and oxidative pressure. Through its effects on Fe-S clusters and Fe(II)/2-oxoglutarate-dependent dioxygenases, NO also couples metabolism to genome stability and epigenetic state: reduced α-KG and increased succinate inhibit TET and Jumonji demethylases, while altered redox and DNIC formation at mononuclear iron centers impose a characteristic “epigenetic lock” with DNA, RNA, and histone methylation patterns that favor oncogenic transcription and nonmutational plasticity. In this way, NO-driven Warburg metabolism directly intersects with the hallmarks of genome instability, epigenetic reprogramming, and phenotypic plasticity that support adaptation and therapy resistance (Fig. 10) [194,195].

The NOS2-COX2-mutant p53 axis provides a particularly clear example of how inflammation, metabolism, and hallmarks of cancer are integrated. This triad activates HIF-1, Akt, and ERK, and reinforcing both “tumor-promoting inflammation” and “avoiding immune destruction” [14]. Spatially, NOS2 and COX2 form clusters that promote metastasis, immunosuppression, and metabolic rewiring, with NOS2 concentrated at the tumor-stroma interface and COX2 showing a similar pattern but extending further into the tumor mass and gradually declining toward the core. In these interface and inner-tumor regions, NO and PGE_2_ drive Warburg metabolism, angiogenesis, and local metastatic traits while simultaneously depleting key nutrients (arginine, glutamine, glucose) and acidifying the TIF [34,196]. These TIF-mediated trans effects impose overlapping layers of immune suppression through arginine depletion, lactate accumulation, altered redox tone, and cytokine skewing that blunt effector T-cell and NK-cell function and favor immunosuppressive myeloid populations, thus directly reinforcing Hanahan's hallmarks of “immune evasion” and “tumor-promoting inflammation” (Fig. 10) [193].

At the single-cell level, quantitative imaging and NO-donor studies show that NO concentrations sufficient to inhibit mitochondrial membrane potential in human breast cancer cells (unpublished data) overlap with those that maximize oncogenic signaling, underscoring that the Warburg phenotype and oncogenic pathway activation are chemically synchronized events rather than parallel coincidences [35]. At early stages of carcinogenesis, NO-driven glycolysis remains partially reversible and mainly serves as a flexible adaptation that supports proliferation and survival under transient stress. With chronic NOS2 activity and inflammatory signaling, however, glycolysis becomes a chemically enforced dependency: irreversible HNO-mediated damage to PDH and other DLD-containing complexes, progressive loss of electron-transport capacity, and epigenetic fixation of HIF-1- and c-Myc-driven transcriptional programs together convert metabolic plasticity into a fixed cellular fate [38]. In Hanahan's updated scheme, this shift exemplifies “unlocking phenotypic plasticity” and “therapy resistance” (Fig. 10). Cells that have undergone NO-imposed Warburg reprogramming are harder to push back into oxidative metabolism, less sensitive to mitochondrial-targeted therapies, and more adept at surviving cytotoxic and immune pressure [193].

Finally, the spatial heterogeneity of NO production and oxygen tension adds a geometric dimension to the hallmarks of cancer. High-flux NO zones near dense NOS2-positive clusters experience nitrosative stress, mitochondrial dysfunction, necrosis, and release of DAMPs that feed back into chronic inflammation and angiogenesis [34]. Intermediate NO levels favor nitrosative signaling without complete toxicity, creating niches enriched for cancer stem-like cells, EMT programs, and metastatic potential. More distal regions from the NO source, where NO falls into the cGMP-signaling range, support angiogenesis and tumor survival with less intense metabolic disruption, completing a mosaic in which different hallmarks dominate in different microdomains of the TME but are all ultimately coordinated by NOS2 activity and NO-driven metabolism (Fig. 10) [35,197,198].

## 14 Conclusions

Taken together, the data discussed in this review position NOS2-derived NO and COX2-derived PGE_2_ as central components of a metabolic-inflammatory circuit that organizes many of the most aggressive features of cancer, driving inflammation, metabolic rewiring, immune evasion, and therapy resistance. Spatially, NOS2-COX2 niches at the tumor-stroma interface and within immune deserts segregate tumors into microdomains with distinct NO levels, oxygen tension, cyclic-nucleotide signaling, and metabolite composition, each enriched for specific hallmarks such as angiogenesis, invasion, cancer stemness, and immune suppression.

Within Hanahan's updated hallmarks of cancer, NOS2-driven Warburg metabolism thus emerges as a systems-level circuit that coordinates deregulated energetics, chronic inflammation, immune escape, genomic/epigenomic instability, and phenotypic plasticity. This integrated view has clear therapeutic implications: disrupting the NOS2-COX2 node, whether by inhibiting NOS2 or COX2 directly, modulating arginine and iron metabolism, or targeting downstream NO-dependent epigenetic and signaling pathways, should not be seen as a single-axis intervention, but as a strategy to simultaneously disrupt multiple, interconnected hallmarks of cancer. As such, rational combinations that inhibit NOS2-COX2 activity while exploiting NO-induced metabolic liabilities (e.g., glutamine addiction, redox stress, ferroptotic susceptibility, and T-cell nutrient competition) may offer a particularly powerful strategy to improve outcomes in NOS2/COX2-high, therapy-refractory tumors.

## Funding

This Research was supported in part by the Cancer Innovation Laboratory, 10.13039/100031022Center for Cancer Research, National Cancer Institute, National Institutes of Health 10.13039/100030692Intramural Research Program project number ZIABC010899-17, ZIABC010300-27 and federal funds from the 10.13039/100000054National Cancer Institute, National Institutes of Health, under contract HHS 75N91019D00024. The contributions of the NIH authors were made as part of their official duties as NIH federal employees, are in compliance with agency policy requirements, and are considered Works of the United States Government. However, the findings and conclusions presented in this paper are those of the author and do not necessarily reflect the views of the NIH or the U.S. Department of Health and Human Services.

## CRediT authorship contribution statement

**Leandro L. Coutinho:** Data curation, Formal analysis, Methodology, Visualization, Writing – review & editing. **Erika M. Palmieri:** Visualization, Writing – review & editing. **Lisa A. Ridnour:** Writing – review & editing. **Robert Y.S. Cheng:** Writing – review & editing. **William F. Heinz:** Writing – review & editing. **Stephen K. Anderson:** Writing – review & editing. **Timothy R. Billiar:** Writing – review & editing. **Jenny Chang:** Writing – review & editing. **Stephen J. Lockett:** Writing – review & editing. **M. Cristina Rangel:** Writing – review & editing. **Douglas D. Thomas:** Writing – review & editing. **Daniel W. McVicar:** Supervision, Writing – review & editing. **David A. Wink:** Conceptualization, Supervision, Writing – original draft, Writing – review & editing.

## Declaration of competing interest

The authors have no potential conflict of interest.

## Data Availability

Data will be made available on request.

## References

[1] Muz B., de la Puente P., Azab F., Azab A.K. (2015). The role of hypoxia in cancer progression, angiogenesis, metastasis, and resistance to therapy. Hypoxia.

[2] Belisario D.C., Kopecka J., Pasino M., Akman M., De Smaele E., Donadelli M. (2020). Hypoxia dictates metabolic rewiring of tumors: implications for chemoresistance. Cells.

[3] Chowdhury M., Das P.K. (2024). Hypoxia: intriguing feature in cancer cell biology. ChemMedChem.

[4] Shang T., Jia Z., Li J., Cao H., Xu H., Cong L. (2025). Unraveling the triad of hypoxia, cancer cell stemness, and drug resistance. J. Hematol. Oncol..

[5] Pandey V.N. (1980). Interdependence of glucose and arginine catabolism in Streptococcus faecalis R. ATCC 8043. Biochem. Biophys. Res. Commun..

[6] Upadhyay M., Samal J., Kandpal M., Singh O.V., Vivekanandan P. (2013). The Warburg effect: insights from the past decade. Pharmacol. Ther..

[7] Potter M., Newport E., Morten K.J. (2016). The Warburg effect: 80 years on. Biochem. Soc. Trans..

[8] Yeung S.J., Pan J., Lee M.H. (2008). Roles of p53, MYC and HIF-1 in regulating glycolysis - the seventh hallmark of cancer. Cell. Mol. Life Sci..

[9] Kaelin W.G. (2011). Cancer and altered metabolism: potential importance of hypoxia-inducible factor and 2-oxoglutarate-dependent dioxygenases. Cold Spring Harb Symp Quant Biol.

[10] Vaupel P., Schmidberger H., Mayer A. (2019). The Warburg effect: essential part of metabolic reprogramming and central contributor to cancer progression. Int. J. Radiat. Biol..

[11] Caneba C.A., Yang L., Baddour J., Curtis R., Win J., Hartig S. (2014). Nitric oxide is a positive regulator of the Warburg effect in ovarian cancer cells. Cell Death Dis..

[12] Chang C.F., Diers A.R., Hogg N. (2015). Cancer cell metabolism and the modulating effects of nitric oxide. Free Radic. Biol. Med..

[13] Coutinho L.L., Femino E.L., Gonzalez A.L., Moffat R.L., Heinz W.F., Cheng R.Y.S. (2024). NOS2 and COX-2 Co-Expression promotes cancer progression: a potential target for developing agents to prevent or treat highly aggressive breast cancer. Int. J. Mol. Sci..

[14] Basudhar D., Glynn S.A., Greer M., Somasundaram V., No J.H., Scheiblin D.A. (2017). Coexpression of NOS2 and COX2 accelerates tumor growth and reduces survival in estrogen receptor-negative breast cancer. Proc Natl Acad Sci U S A..

[15] Liberti M.V., Locasale J.W. (2016). The warburg effect: how does it benefit cancer cells?. Trends Biochem. Sci..

[16] Lu J., Tan M., Cai Q. (2015). The Warburg effect in tumor progression: mitochondrial oxidative metabolism as an anti-metastasis mechanism. Cancer Lett..

[17] Thomas D.D., Ridnour L.A., Isenberg J.S., Flores-Santana W., Switzer C.H., Donzelli S. (2008). The chemical biology of nitric oxide: implications in cellular signaling. Free Radic. Biol. Med..

[18] Thomas D.D., Heinecke J.L., Ridnour L.A., Cheng R.Y., Kesarwala A.H., Switzer C.H. (2015). Signaling and stress: the redox landscape in NOS2 biology. Free Radic. Biol. Med..

[19] Billiar T.R., Curran R.D., Harbrecht B.G., Stadler J., Williams D.L., Ochoa J.B. (1992). Association between synthesis and release of cGMP and nitric oxide biosynthesis by hepatocytes. Am. J. Physiol..

[20] Salimian Rizi B., Achreja A., Nagrath D. (2017). Nitric oxide: the forgotten child of tumor metabolism. Trends Cancer.

[21] Lopez-Sanchez L.M., Aranda E., Rodriguez-Ariza A. (2020). Nitric oxide and tumor metabolic reprogramming. Biochem. Pharmacol..

[22] Drapier J.C., Hibbs J.B. (1986). Murine cytotoxic activated macrophages inhibit aconitase in tumor cells. Inhibition involves the iron-sulfur prosthetic group and is reversible. J. Clin. Investig..

[23] Stuehr D.J., Nathan C.F. (1989). Nitric oxide. A macrophage product responsible for cytostasis and respiratory inhibition in tumor target cells. J. Exp. Med..

[24] Hibbs J.B., Westenfelder C., Taintor R., Vavrin Z., Kablitz C., Baranowski R.L. (1992). Evidence for cytokine-inducible nitric oxide synthesis from L-arginine in patients receiving interleukin-2 therapy. J. Clin. Investig..

[25] Stuehr D.J., Marletta M.A. (1985). Mammalian nitrate biosynthesis: mouse macrophages produce nitrite and nitrate in response to Escherichia coli lipopolysaccharide. Proc Natl Acad Sci U S A..

[26] Iyengar R., Stuehr D.J., Marletta M.A. (1987). Macrophage synthesis of nitrite, nitrate, and N-nitrosamines: precursors and role of the respiratory burst. Proc. Natl. Acad. Sci. U. S. A..

[27] Miwa M., Stuehr D.J., Marletta M.A., Wishnok J.S., Tannenbaum S.R. (1987). Nitrosation of amines by stimulated macrophages. Carcinogenesis.

[28] Stuehr D.J., Marletta M.A. (1987). Synthesis of nitrite and nitrate in murine macrophage cell lines. Cancer Res..

[29] Ruttimann J. (2007). Macrophages and nitric oxide: a deadly combination. J. Exp. Med..

[30] Heinecke J.L., Ridnour L.A., Cheng R.Y., Switzer C.H., Lizardo M.M., Khanna C. (2014). Tumor microenvironment-based feed-forward regulation of NOS2 in breast cancer progression. Proc. Natl. Acad. Sci. U. S. A..

[31] Li L., Zhu L., Hao B., Gao W., Wang Q., Li K. (2017). iNOS-derived nitric oxide promotes glycolysis by inducing pyruvate kinase M2 nuclear translocation in ovarian cancer. Oncotarget.

[32] Alam A., Smith S.C., Gobalakrishnan S., McGinn M., Yakovlev V.A., Rabender C.S. (2023). Uncoupled nitric oxide synthase activity promotes colorectal cancer progression. Front. Oncol..

[33] Zhang L., Ren B.C., Wei F., Liu Y., Gao Y., Yuan B. (2023). Ferroptosis regulator NOS2 is closely associated with the prognosis and cell malignant behaviors of hepatoblastoma: a bioinformatic and in vitro study. Front. Oncol..

[34] Ridnour L.A., Cheng R.Y., Heinz W.F., Pore M., Gonzalez A.L., Femino E.L. (2025). Elevated tumor NOS2/COX2 promotes immunosuppressive phenotypes associated with poor survival in ER- breast cancer. JCI Insight.

[35] Somasundaram V., Basudhar D., Bharadwaj G., No J.H., Ridnour L.A., Cheng R.Y.S. (2019). Molecular mechanisms of nitric oxide in cancer progression, signal transduction, and metabolism. Antioxid Redox Signal.

[36] Cheng R.Y.S., Ridnour L.A., Wink A.L., Gonzalez A.L., Femino E.L., Rittscher H. (2023). Interferon-gamma is quintessential for NOS2 and COX2 expression in ER(-) breast tumors that lead to poor outcome. Cell Death Dis..

[37] Takabayashi A., Kawai Y., Iwata S., Kanai M., Denno R., Kawada K. (2000). Nitric oxide induces a decrease in the mitochondrial membrane potential of peripheral blood lymphocytes, especially in natural killer cells. Antioxid Redox Signal.

[38] Palmieri E.M., Holewinski R., McGinity C.L., Pierri C.L., Maio N., Weiss J.M. (2023). Pyruvate dehydrogenase operates as an intramolecular nitroxyl generator during macrophage metabolic reprogramming. Nat. Commun..

[39] Rizza S., Di Leo L., Mandatori S., De Zio D., Filomeni G. (2020). Mitophagy contributes to alpha-tocopheryl succinate toxicity in GSNOR-deficient hepatocellular carcinoma. Biochem. Pharmacol..

[40] Lazzarino G., Amorini A.M., Signoretti S., Musumeci G., Lazzarino G., Caruso G. (2019). Pyruvate dehydrogenase and tricarboxylic acid cycle enzymes are sensitive targets of traumatic brain injury induced metabolic derangement. Int. J. Mol. Sci..

[41] Palmieri E.M., Gonzalez-Cotto M., Baseler W.A., Davies L.C., Ghesquiere B., Maio N. (2020). Nitric oxide orchestrates metabolic rewiring in M1 macrophages by targeting aconitase 2 and pyruvate dehydrogenase. Nat. Commun..

[42] Burwell L.S., Nadtochiy S.M., Tompkins A.J., Young S., Brookes P.S. (2006). Direct evidence for S-nitrosation of mitochondrial complex I. Biochem. J..

[43] Baseler W.A., Davies L.C., Quigley L., Ridnour L.A., Weiss J.M., Hussain S.P. (2016). Autocrine IL-10 functions as a rheostat for M1 macrophage glycolytic commitment by tuning nitric oxide production. Redox Biol..

[44] Aguirre E., Rodriguez-Juarez F., Bellelli A., Gnaiger E., Cadenas S. (2010). Kinetic model of the inhibition of respiration by endogenous nitric oxide in intact cells. Biochim. Biophys. Acta.

[45] Silva A.J.C., de Lavor M.S.L. (2025). Nitroxidative stress, cell-signaling pathways, and manganese porphyrins: therapeutic potential in neuropathic pain. Int. J. Mol. Sci..

[46] Costa N.J., Dahm C.C., Hurrell F., Taylor E.R., Murphy M.P. (2003). Interactions of mitochondrial thiols with nitric oxide. Antioxid Redox Signal.

[47] Borutaite V., Morkuniene R., Arandarcikaite O., Jekabsone A., Barauskaite J., Brown G.C. (2009). Nitric oxide protects the heart from ischemia-induced apoptosis and mitochondrial damage via protein kinase G mediated blockage of permeability transition and cytochrome c release. J. Biomed. Sci..

[48] Kennedy M.C., Emptage M.H., Dreyer J.L., Beinert H. (1983). The role of iron in the activation-inactivation of aconitase. J. Biol. Chem..

[49] Bailey J.D., Diotallevi M., Nicol T., McNeill E., Shaw A., Chuaiphichai S. (2019). Nitric oxide modulates metabolic remodeling in inflammatory macrophages through TCA cycle regulation and itaconate accumulation. Cell Rep..

[50] Palmieri E.M., McGinity C., Wink D.A., McVicar D.W. (2020). Nitric oxide in macrophage immunometabolism: hiding in Plain sight. Metabolites.

[51] Miranda K.M., Paolocci N., Katori T., Thomas D.D., Ford E., Bartberger M.D. (2003). A biochemical rationale for the discrete behavior of nitroxyl and nitric oxide in the cardiovascular system. Proc Natl Acad Sci U S A..

[52] Bianco C.L., Toscano J.P., Bartberger M.D., Fukuto J.M. (2017). The chemical biology of HNO signaling. Arch. Biochem. Biophys..

[53] Reed L.J., Hackert M.L. (1990). Structure-function relationships in dihydrolipoamide acyltransferases. J. Biol. Chem..

[54] Selak M.A., Armour S.M., MacKenzie E.D., Boulahbel H., Watson D.G., Mansfield K.D. (2005). Succinate links TCA cycle dysfunction to oncogenesis by inhibiting HIF-alpha prolyl hydroxylase. Cancer Cell.

[55] MacKenzie E.D., Selak M.A., Tennant D.A., Payne L.J., Crosby S., Frederiksen C.M. (2007). Cell-permeating alpha-ketoglutarate derivatives alleviate pseudohypoxia in succinate dehydrogenase-deficient cells. Mol. Cell Biol..

[56] Infantino V., Convertini P., Cucci L., Panaro M.A., Di Noia M.A., Calvello R. (2011). The mitochondrial citrate carrier: a new player in inflammation. Biochem. J..

[57] Tannahill G.M., Curtis A.M., Adamik J., Palsson-McDermott E.M., McGettrick A.F., Goel G. (2013). Succinate is an inflammatory signal that induces IL-1beta through HIF-1alpha. Nature.

[58] Everts B., Amiel E., Huang S.C., Smith A.M., Chang C.H., Lam W.Y. (2014). TLR-driven early glycolytic reprogramming via the kinases TBK1-IKKvarepsilon supports the anabolic demands of dendritic cell activation. Nat. Immunol..

[59] Liu P.S., Wang H., Li X., Chao T., Teav T., Christen S. (2017). alpha-ketoglutarate orchestrates macrophage activation through metabolic and epigenetic reprogramming. Nat. Immunol..

[60] Cleeter M.W., Cooper J.M., Darley-Usmar V.M., Moncada S., Schapira A.H. (1994). Reversible inhibition of cytochrome c oxidase, the terminal enzyme of the mitochondrial respiratory chain, by nitric oxide. Implications for neurodegenerative diseases. FEBS Lett..

[61] Brown G.C., Cooper C.E. (1994). Nanomolar concentrations of nitric oxide reversibly inhibit synaptosomal respiration by competing with oxygen at cytochrome oxidase. FEBS Lett..

[62] Schweizer M., Richter C. (1994). Nitric oxide potently and reversibly deenergizes mitochondria at low oxygen tension. Biochem. Biophys. Res. Commun..

[63] Radi R., Rodriguez M., Castro L., Telleri R. (1994). Inhibition of mitochondrial electron transport by peroxynitrite. Arch. Biochem. Biophys..

[64] Jourd'heuil D., Jourd'heuil F.L., Kutchukian P.S., Musah R.A., Wink D.A., Grisham M.B. (2001). Reaction of superoxide and nitric oxide with peroxynitrite. Implications for peroxynitrite-mediated oxidation reactions in vivo. J. Biol. Chem..

[65] Thomas D.D., Ridnour L.A., Espey M.G., Donzelli S., Ambs S., Hussain S.P. (2006). Superoxide fluxes limit nitric oxide-induced signaling. J. Biol. Chem..

[66] Candas D., Li J.J. (2014). MnSOD in oxidative stress response-potential regulation via mitochondrial protein influx. Antioxid Redox Signal.

[67] Chavez M.D., Lakshmanan N., Kavdia M. (2007). Impact of superoxide dismutase on nitric oxide and peroxynitrite levels in the microcirculation--a computational model. Annu Int Conf IEEE Eng Med Biol Soc.

[68] Holley A.K., Bakthavatchalu V., Velez-Roman J.M., St Clair D.K. (2011). Manganese superoxide dismutase: guardian of the powerhouse. Int. J. Mol. Sci..

[69] Kornberg M.D., Sen N., Hara M.R., Juluri K.R., Nguyen J.V., Snowman A.M. (2010). GAPDH mediates nitrosylation of nuclear proteins. Nat. Cell Biol..

[70] Almeida A., Almeida J., Bolanos J.P., Moncada S. (2001). Different responses of astrocytes and neurons to nitric oxide: the role of glycolytically generated ATP in astrocyte protection. Proc Natl Acad Sci U S A..

[71] Krawczyk C.M., Holowka T., Sun J., Blagih J., Amiel E., DeBerardinis R.J. (2010). Toll-like receptor-induced changes in glycolytic metabolism regulate dendritic cell activation. Blood.

[72] Everts B., Amiel E., van der Windt G.J., Freitas T.C., Chott R., Yarasheski K.E. (2012). Commitment to glycolysis sustains survival of NO-producing inflammatory dendritic cells. Blood.

[73] Thwe P.M., Amiel E. (2018). The role of nitric oxide in metabolic regulation of dendritic cell immune function. Cancer Lett..

[74] Picco N., Gatenby R.A., Anderson A.R.A. (2017). Stem cell plasticity and niche dynamics in cancer progression. IEEE Trans. Biomed. Eng..

[75] Dobbins R.L., Szczepaniak L.S., Bentley B., Esser V., Myhill J., McGarry J.D. (2001). Prolonged inhibition of muscle carnitine palmitoyltransferase-1 promotes intramyocellular lipid accumulation and insulin resistance in rats. Diabetes.

[76] Boren J., Brindle K.M. (2012). Apoptosis-induced mitochondrial dysfunction causes cytoplasmic lipid droplet formation. Cell Death Differ..

[77] Zirath H., Frenzel A., Oliynyk G., Segerstrom L., Westermark U.K., Larsson K. (2013). MYC inhibition induces metabolic changes leading to accumulation of lipid droplets in tumor cells. Proc Natl Acad Sci U S A..

[78] Rosas-Ballina M., Guan X.L., Schmidt A., Bumann D. (2020). Classical Activation of Macrophages Leads to Lipid Droplet Formation Without de novo Fatty Acid Synthesis. Front. Immunol..

[79] Choi M.S., Jung J.Y., Kim H.J., Ham M.R., Lee T.R., Shin D.W. (2016). S-nitrosylation of fatty acid synthase regulates its activity through dimerization. J. Lipid Res..

[80] Metzen E., Zhou J., Jelkmann W., Fandrey J., Brune B. (2003). Nitric oxide impairs normoxic degradation of HIF-1alpha by inhibition of prolyl hydroxylases. Mol. Biol. Cell.

[81] DeMichele E., Buret A.G., Taylor C.T. (2024). Hypoxia-inducible factor-driven glycolytic adaptations in host-microbe interactions. Pflugers Arch..

[82] Wang J., Yang P., Yu T., Gao M., Liu D., Zhang J. (2022). Lactylation of PKM2 suppresses inflammatory metabolic adaptation in pro-inflammatory macrophages. Int. J. Biol. Sci..

[83] Demaria M., Poli V. (2012). PKM2, STAT3 and HIF-1alpha: the Warburg's vicious circle. JAK-STAT.

[84] Switzer C.H., Glynn S.A., Cheng R.Y., Ridnour L.A., Green J.E., Ambs S. (2012). S-nitrosylation of EGFR and Src activates an oncogenic signaling network in human basal-like breast cancer. Mol. Cancer Res..

[85] Thong F.S., Bilan P.J., Klip A. (2007). The Rab GTPase-activating protein AS160 integrates Akt, protein kinase C, and AMP-activated protein kinase signals regulating GLUT4 traffic. Diabetes.

[86] Robey R.B., Hay N. (2009). Is Akt the "Warburg kinase"?-Akt-energy metabolism interactions and oncogenesis. Semin. Cancer Biol..

[87] Switzer C.H., Cheng R.Y., Ridnour L.A., Glynn S.A., Ambs S., Wink D.A. (2012). Ets-1 is a transcriptional mediator of oncogenic nitric oxide signaling in estrogen receptor-negative breast cancer. Breast Cancer Res..

[88] Yang W., Zheng Y., Xia Y., Ji H., Chen X., Guo F. (2012). ERK1/2-dependent phosphorylation and nuclear translocation of PKM2 promotes the Warburg effect. Nat. Cell Biol..

[89] Papa S., Choy P.M., Bubici C. (2019). The ERK and JNK pathways in the regulation of metabolic reprogramming. Oncogene.

[90] Kawashima I., Mitsumori T., Nozaki Y., Yamamoto T., Shobu-Sueki Y., Nakajima K. (2015). Negative regulation of the LKB1/AMPK pathway by ERK in human acute myeloid leukemia cells. Exp. Hematol..

[91] Chang C.W., Chen Y.S., Tsay Y.G., Han C.L., Chen Y.J., Yang C.C. (2018). ROS-independent ER stress-mediated NRF2 activation promotes warburg effect to maintain stemness-associated properties of cancer-initiating cells. Cell Death Dis..

[92] Vodovotz Y., Chesler L., Chong H., Kim S.J., Simpson J.T., DeGraff W. (1999). Regulation of transforming growth factor beta1 by nitric oxide. Cancer Res..

[93] Hua W., Ten Dijke P., Kostidis S., Giera M., Hornsveld M. (2020). TGFbeta-induced metabolic reprogramming during epithelial-to-mesenchymal transition in cancer. Cell. Mol. Life Sci..

[94] Huang Y., Chen Z., Lu T., Bi G., Li M., Liang J. (2021). HIF-1alpha switches the functionality of TGF-beta signaling via changing the partners of smads to drive glucose metabolic reprogramming in non-small cell lung cancer. J. Exp. Clin. Cancer Res..

[95] Angioni R., Sanchez-Rodriguez R., Viola A., Molon B. (2021). TGF-beta in cancer: Metabolic driver of the tolerogenic crosstalk in the tumor microenvironment. Cancers (Basel).

[96] Liu H., Chen Y.G. (2022). The interplay between TGF-beta signaling and cell metabolism. Front. Cell Dev. Biol..

[97] Bensaad K., Tsuruta A., Selak M.A., Vidal M.N., Nakano K., Bartrons R. (2006). TIGAR, a p53-inducible regulator of glycolysis and apoptosis. Cell.

[98] Moulder D.E., Hatoum D., Tay E., Lin Y., McGowan E.M. (2018). The roles of p53 in mitochondrial dynamics and cancer metabolism: the pendulum between survival and death in breast cancer?. Cancers (Basel).

[99] Said R., Hong D.S., Warneke C.L., Lee J.J., Wheler J.J., Janku F. (2013). P53 mutations in advanced cancers: clinical characteristics, outcomes, and correlation between progression-free survival and bevacizumab-containing therapy. Oncotarget.

[100] Messmer U.K., Brune B. (1996). Nitric oxide-induced apoptosis: p53-dependent and p53-independent signalling pathways. Biochem. J..

[101] Rath M., Muller I., Kropf P., Closs E.I., Munder M. (2014). Metabolism via arginase or nitric oxide synthase: two competing arginine pathways in macrophages. Front. Immunol..

[102] Kan M.J., Lee J.E., Wilson J.G., Everhart A.L., Brown C.M., Hoofnagle A.N. (2015). Arginine deprivation and immune suppression in a mouse model of Alzheimer's disease. J. Neurosci..

[103] Mori M., Gotoh T. (2000). Regulation of nitric oxide production by arginine metabolic enzymes. Biochem. Biophys. Res. Commun..

[104] Wu G., Morris S.M. (1998). Arginine metabolism: nitric oxide and beyond. Biochem. J..

[105] Iniesta V., Gomez-Nieto L.C., Corraliza I. (2001). The inhibition of arginase by N(omega)-hydroxy-l-arginine controls the growth of Leishmania inside macrophages. J. Exp. Med..

[106] Lasch M., Caballero-Martinez A., Troidl K., Schloegl I., Lautz T., Deindl E. (2016). Arginase inhibition attenuates arteriogenesis and interferes with M2 macrophage accumulation. Lab. Invest..

[107] Ridnour L.A., Heinz W.F., Cheng R.Y.S., Wink A.L., Kedei N., Pore M. (2024). Tumor NOS2 and COX2 spatial juxtaposition with CD8+ T cells promote metastatic and cancer stem cell niches that lead to poor outcome in ER- breast cancer. Cancer Res. Commun..

[108] Grzybowski M.M., Ucal Y., Muchowicz A., Rejczak T., Kikulska A., Gluchowska K.M. (2025). Metabolomic reprogramming of the tumor microenvironment by dual arginase inhibitor OATD-02 boosts anticancer immunity. Sci. Rep..

[109] Hesterberg R.S., Cleveland J.L., Epling-Burnette P.K. (2018). Role of polyamines in immune cell functions. Med. Sci..

[110] Pfeifhofer-Obermair C., Tymoszuk P., Petzer V., Weiss G., Nairz M. (2018). Iron in the tumor microenvironment-connecting the dots. Front. Oncol..

[111] Castro L., Tortora V., Mansilla S., Radi R. (2019). Aconitases: Non-redox iron-sulfur proteins sensitive to reactive species. Acc. Chem. Res..

[112] Toledo J.C., Bosworth C.A., Hennon S.W., Mahtani H.A., Bergonia H.A., Lancaster J.R. (2008). Nitric oxide-induced conversion of cellular chelatable iron into macromolecule-bound paramagnetic dinitrosyliron complexes. J. Biol. Chem..

[113] Hickok J.R., Sahni S., Shen H., Arvind A., Antoniou C., Fung L.W. (2011). Dinitrosyliron complexes are the most abundant nitric oxide-derived cellular adduct: biological parameters of assembly and disappearance. Free Radic. Biol. Med..

[114] Hickok J.R., Vasudevan D., Thatcher G.R., Thomas D.D. (2012). Is S-nitrosocysteine a true surrogate for nitric oxide?. Antioxid Redox Signal.

[115] Bouton C., Drapier J.C. (2003). Iron regulatory proteins as NO signal transducers. Sci. STKE.

[116] Bogdan A.R., Miyazawa M., Hashimoto K., Tsuji Y. (2016). Regulators of iron homeostasis: new players in metabolism, cell death, and disease. Trends Biochem. Sci..

[117] Russo M., Gualdrini F., Vallelonga V., Prosperini E., Noberini R., Pedretti S. (2024). Acetyl-CoA production by Mediator-bound 2-ketoacid dehydrogenases boosts de novo histone acetylation and is regulated by nitric oxide. Mol Cell.

[118] Hickok J.R., Vasudevan D., Antholine W.E., Thomas D.D. (2013). Nitric oxide modifies global histone methylation by inhibiting Jumonji C domain-containing demethylases. J. Biol. Chem..

[119] Palczewski M.B., Kuschman H.P., Hoffman B.M., Kathiresan V., Yang H., Glynn S.A. (2025). Nitric oxide inhibits ten-eleven translocation DNA demethylases to regulate 5mC and 5hmC across the genome. Nat. Commun..

[120] Kuschman H.P., Palczewski M.B., Hoffman B., Menhart M., Wang X., Glynn S. (2023). Nitric oxide inhibits FTO demethylase activity to regulate N(6)-methyladenosine mRNA methylation. Redox Biol..

[121] Vasudevan D., Hickok J.R., Bovee R.C., Pham V., Mantell L.L., Bahroos N. (2015). Nitric oxide regulates gene expression in cancers by controlling histone posttranslational modifications. Cancer Res..

[122] Thomas D.D., Espey M.G., Ridnour L.A., Hofseth L.J., Mancardi D., Harris C.C. (2004). Hypoxic inducible factor 1alpha, extracellular signal-regulated kinase, and p53 are regulated by distinct threshold concentrations of nitric oxide. Proc Natl Acad Sci U S A..

[123] Hickok J.R., Sahni S., Mikhed Y., Bonini M.G., Thomas D.D. (2011). Nitric oxide suppresses tumor cell migration through N-Myc downstream-regulated gene-1 (NDRG1) expression: role of chelatable iron. J. Biol. Chem..

[124] Kovacevic Z., Menezes S.V., Sahni S., Kalinowski D.S., Bae D.H., Lane D.J. (2016). The metastasis suppressor, N-MYC downstream-regulated Gene-1 (NDRG1), down-regulates the ErbB family of receptors to inhibit downstream oncogenic signaling pathways. J. Biol. Chem..

[125] Kovacevic Z., Sivagurunathan S., Mangs H., Chikhani S., Zhang D., Richardson D.R. (2011). The metastasis suppressor, N-myc downstream regulated gene 1 (NDRG1), upregulates p21 via p53-independent mechanisms. Carcinogenesis.

[126] Azad M.G., Russell T.M., Gu X., Zhao X., Richardson V., Wijesinghe T.P. (2025). NDRG1 and its family members: more than just metastasis suppressor proteins and targets of thiosemicarbazones. J. Biol. Chem..

[127] Montfort W.R., Wales J.A., Weichsel A. (2017). Structure and activation of soluble guanylyl cyclase, the nitric oxide sensor. Antioxid Redox Signal.

[128] Liu R., Kang Y., Chen L. (2021). Activation mechanism of human soluble guanylate cyclase by stimulators and activators. Nat. Commun..

[129] Collman J.P., Dey A., Decreau R.A., Yang Y., Hosseini A., Solomon E.I. (2008). Interaction of nitric oxide with a functional model of cytochrome c oxidase. Proc. Natl. Acad. Sci. U. S. A..

[130] Kalous K.S., Wynia-Smith S.L., Summers S.B., Smith B.C. (2020). Human sirtuins are differentially sensitive to inhibition by nitrosating agents and other cysteine oxidants. J. Biol. Chem..

[131] Kalous K.S., Wynia-Smith S.L., Smith B.C. (2021). Sirtuin oxidative post-translational modifications. Front. Physiol..

[132] Mengel A., Ageeva A., Georgii E., Bernhardt J., Wu K., Durner J. (2017). Nitric oxide modulates histone acetylation at stress genes by inhibition of histone deacetylases. Plant Physiol..

[133] Salvatori L., Spallotta F., Gaetano C., Illi B. (2021). Pillars and gaps of S-Nitrosylation-Dependent epigenetic regulation in physiology and cancer. Life.

[134] Rubio K., Hernandez-Cruz E.Y., Rogel-Ayala D.G., Sarvari P., Isidoro C., Barreto G. (2023). Nutriepigenomics in environmental-associated oxidative stress. Antioxidants.

[135] Sahni S., Hickok J.R., Thomas D.D. (2018). Nitric oxide reduces oxidative stress in cancer cells by forming dinitrosyliron complexes. Nitric Oxide.

[136] Hickok J.R., Vasudevan D., Jablonski K., Thomas D.D. (2013). Oxygen dependence of nitric oxide-mediated signaling. Redox Biol..

[137] Thomas D.D. (2015). Breathing new life into nitric oxide signaling: a brief overview of the interplay between oxygen and nitric oxide. Redox Biol..

[138] Somasundaram V., Gilmore A.C., Basudhar D., Palmieri E.M., Scheiblin D.A., Heinz W.F. (2020). Inducible nitric oxide synthase-derived extracellular nitric oxide flux regulates proinflammatory responses at the single cell level. Redox Biol..

[139] Gross T.J., Kremens K., Powers L.S., Brink B., Knutson T., Domann F.E. (2014). Epigenetic silencing of the human NOS2 gene: rethinking the role of nitric oxide in human macrophage inflammatory responses. J. Immunol..

[140] Vitek M.P., Brown C., Xu Q., Dawson H., Mitsuda N., Colton C.A. (2006). Characterization of NO and cytokine production in immune-activated microglia and peritoneal macrophages derived from a mouse model expressing the human NOS2 gene on a mouse NOS2 knockout background. Antioxid Redox Signal.

[141] Rengasamy A., Johns R.A. (1996). Determination of Km for oxygen of nitric oxide synthase isoforms. J Pharmacol Exp Ther.

[142] Abu-Soud H.M., Ichimori K., Nakazawa H., Stuehr D.J. (2001). Regulation of inducible nitric oxide synthase by self-generated NO. Biochemistry.

[143] Hoos M.D., Vitek M.P., Ridnour L.A., Wilson J., Jansen M., Everhart A. (2014). The impact of human and mouse differences in NOS2 gene expression on the brain's redox and immune environment. Mol. Neurodegener..

[144] Diaz-Guerra M.J., Velasco M., Martin-Sanz P., Bosca L. (1996). Evidence for common mechanisms in the transcriptional control of type II nitric oxide synthase in isolated hepatocytes. Requirement of NF-kappaB activation after stimulation with bacterial cell wall products and phorbol esters. J. Biol. Chem..

[145] Ganster R.W., Taylor B.S., Shao L., Geller D.A. (2001). Complex regulation of human inducible nitric oxide synthase gene transcription by Stat 1 and NF-kappa B. Proc Natl Acad Sci U S A..

[146] Xu W., Comhair S.A., Zheng S., Chu S.C., Marks-Konczalik J., Moss J. (2003). STAT-1 and c-Fos interaction in nitric oxide synthase-2 gene activation. Am. J. Physiol. Lung Cell. Mol. Physiol..

[147] Farlik M., Reutterer B., Schindler C., Greten F., Vogl C., Muller M. (2010). Nonconventional initiation complex assembly by STAT and NF-kappaB transcription factors regulates nitric oxide synthase expression. Immunity.

[148] Du Q., Zhang X., Liu Q., Zhang X., Bartels C.E., Geller D.A. (2013). Nitric oxide production upregulates Wnt/beta-catenin signaling by inhibiting Dickkopf-1. Cancer Res..

[149] Du Q., Park K.S., Guo Z., He P., Nagashima M., Shao L. (2006). Regulation of human nitric oxide synthase 2 expression by Wnt beta-catenin signaling. Cancer Res..

[150] Naugler W.E., Karin M. (2008). NF-kappaB and cancer-identifying targets and mechanisms. Curr. Opin. Genet. Dev..

[151] Gilmore A.C., Flaherty S.J., Somasundaram V., Scheiblin D.A., Lockett S.J., Wink D.A. (2021). An in vitro tumorigenesis model based on live-cell-generated oxygen and nutrient gradients. Commun. Biol..

[152] Xing F., Luan Y., Cai J., Wu S., Mai J., Gu J. (2017). The anti-warburg effect elicited by the cAMP-PGC1alpha pathway drives differentiation of glioblastoma cells into astrocytes. Cell Rep..

[153] Strickland M., Stoll E.A. (2017). Metabolic reprogramming in Glioma. Front. Cell Dev. Biol..

[154] Aslam M., Ladilov Y. (2021). Regulation of mitochondrial homeostasis by sAC-Derived cAMP pool: basic and translational aspects. Cells.

[155] Bhalla K., Hwang B.J., Dewi R.E., Ou L., Twaddel W., Fang H.B. (2011). PGC1alpha promotes tumor growth by inducing gene expression programs supporting lipogenesis. Cancer Res..

[156] O'Callaghan G., Houston A. (2015). Prostaglandin E2 and the EP receptors in malignancy: possible therapeutic targets?. Br. J. Pharmacol..

[157] Liao M., Yao D., Wu L., Luo C., Wang Z., Zhang J. (2024). Targeting the Warburg effect: a revisited perspective from molecular mechanisms to traditional and innovative therapeutic strategies in cancer. Acta Pharm. Sin. B.

[158] Zhang H., Liu Y., Liu J., Chen J., Wang J., Hua H. (2024). cAMP-PKA/EPAC signaling and cancer: the interplay in tumor microenvironment. J. Hematol. Oncol..

[159] Paduch R., Kandefer-Szerszen M. (2011). Nitric Oxide (NO) and Cyclooxygenase-2 (COX-2) cross-talk in Co-Cultures of tumor spheroids with normal cells. Cancer Microenviron.

[160] Fernandez-Marcos P.J., Auwerx J. (2011). Regulation of PGC-1alpha, a nodal regulator of mitochondrial biogenesis. Am. J. Clin. Nutr..

[161] Martins Pinto M., Paumard P., Bouchez C., Ransac S., Duvezin-Caubet S., Mazat J.P. (2023). The Warburg effect and mitochondrial oxidative phosphorylation: friends or foes?. Biochim. Biophys. Acta Bioenerg..

[162] Wilde L., Roche M., Domingo-Vidal M., Tanson K., Philp N., Curry J. (2017). Metabolic coupling and the Reverse Warburg Effect in cancer: implications for novel biomarker and anticancer agent development. Semin. Oncol..

[163] Liang L., Li W., Li X., Jin X., Liao Q., Li Y. (2022). 'Reverse Warburg effect' of cancer-associated fibroblasts. Int. J. Oncol..

[164] Pavlides S., Vera I., Gandara R., Sneddon S., Pestell R.G., Mercier I. (2012). Warburg meets autophagy: cancer-associated fibroblasts accelerate tumor growth and metastasis via oxidative stress, mitophagy, and aerobic glycolysis. Antioxid Redox Signal.

[165] Han J., Meng Q., Xi Q., Zhang Y., Zhuang Q., Han Y. (2016). Interleukin-6 stimulates aerobic glycolysis by regulating PFKFB3 at early stage of colorectal cancer. Int. J. Oncol..

[166] Fousek K., Horn L.A., Palena C. (2021). Interleukin-8: a chemokine at the intersection of cancer plasticity, angiogenesis, and immune suppression. Pharmacol. Ther..

[167] Qiao Q., Hu S., Wang X. (2024). The regulatory roles and clinical significance of glycolysis in tumor. Cancer Commun..

[168] Salmiheimo A., Mustonen H., Vainionpaa S., Shen Z., Kemppainen E., Puolakkainen P. (2017). Tumour-associated macrophages activate migration and STAT3 in pancreatic ductal adenocarcinoma cells in co-cultures. Pancreatology.

[169] Zhong Z., Yang K., Li Y., Zhou S., Yao H., Zhao Y. (2024). Tumor-associated macrophages drive glycolysis through the IL-8/STAT3/GLUT3 signaling pathway in pancreatic cancer progression. Cancer Lett..

[170] Martinez-Outschoorn U.E., Pestell R.G., Howell A., Tykocinski M.L., Nagajyothi F., Machado F.S. (2011). Energy transfer in "parasitic" cancer metabolism: mitochondria are the powerhouse and Achilles' heel of tumor cells. Cell Cycle.

[171] Song Y.C., Lee S.E., Jin Y., Park H.W., Chun K.H., Lee H.W. (2020). Classifying the linkage between adipose tissue inflammation and tumor growth through cancer-associated adipocytes. Mol. Cells.

[172] Nagasaki T., Hara M., Nakanishi H., Takahashi H., Sato M., Takeyama H. (2014). Interleukin-6 released by colon cancer-associated fibroblasts is critical for tumour angiogenesis: anti-interleukin-6 receptor antibody suppressed angiogenesis and inhibited tumour-stroma interaction. Br. J. Cancer.

[173] Zhao Y., Shen M., Wu L., Yang H., Yao Y., Yang Q. (2023). Stromal cells in the tumor microenvironment: accomplices of tumor progression?. Cell Death Dis..

[174] Isenberg J.S., Ridnour L.A., Perruccio E.M., Espey M.G., Wink D.A., Roberts D.D. (2005). Thrombospondin-1 inhibits endothelial cell responses to nitric oxide in a cGMP-dependent manner. Proc Natl Acad Sci U S A..

[175] Ridnour L.A., Isenberg J.S., Espey M.G., Thomas D.D., Roberts D.D., Wink D.A. (2005). Nitric oxide regulates angiogenesis through a functional switch involving thrombospondin-1. Proc. Natl. Acad. Sci. U. S. A..

[176] Murphy-Ullrich J.E., Suto M.J. (2018). Thrombospondin-1 regulation of latent TGF-beta activation: a therapeutic target for fibrotic disease. Matrix Biol..

[177] Lala P.K., Chakraborty C. (2001). Role of nitric oxide in carcinogenesis and tumour progression. Lancet Oncol..

[178] Gallo O., Schiavone N., Papucci L., Sardi I., Magnelli L., Franchi A. (2003). Down-regulation of nitric oxide synthase-2 and cyclooxygenase-2 pathways by p53 in squamous cell carcinoma. Am. J. Pathol..

[179] Forrester K., Ambs S., Lupold S.E., Kapust R.B., Spillare E.A., Weinberg W.C. (1996). Nitric oxide-induced p53 accumulation and regulation of inducible nitric oxide synthase expression by wild-type p53. Proc. Natl. Acad. Sci. U. S. A..

[180] Dave B., Gonzalez D.D., Liu Z.B., Li X., Wong H., Granados S. (2017). Role of RPL39 in metaplastic breast cancer. J Natl Cancer Inst.

[181] Cheng R.Y.S., Burkett S., Ambs S., Moody T., Wink D.A., Ridnour L.A. (2023). Chronic exposure to nitric oxide induces P53 mutations and malignant-like features in human breast epithelial cells. Biomolecules.

[182] Llibre A., Kucuk S., Gope A., Certo M., Mauro C. (2025). Lactate: a key regulator of the immune response. Immunity.

[183] Fletcher M., Ramirez M.E., Sierra R.A., Raber P., Thevenot P., Al-Khami A.A. (2015). l-Arginine depletion blunts antitumor T-cell responses by inducing myeloid-derived suppressor cells. Cancer Res..

[184] Oh M.H., Sun I.H., Zhao L., Leone R.D., Sun I.M., Xu W. (2020). Targeting glutamine metabolism enhances tumor-specific immunity by modulating suppressive myeloid cells. J. Clin. Investig..

[185] Elia I., Haigis M.C. (2021). Metabolites and the tumour microenvironment: from cellular mechanisms to systemic metabolism. Nat. Metab..

[186] Parkinson E.K., Adamski J., Zahn G., Gaumann A., Flores-Borja F., Ziegler C. (2021). Extracellular citrate and metabolic adaptations of cancer cells. Cancer Metastasis Rev..

[187] Zhang X.D., Qin Z.H., Wang J. (2010). The role of p53 in cell metabolism. Acta Pharmacol. Sin..

[188] Ambs S., Hussain S.P., Harris C.C. (1997). Interactive effects of nitric oxide and the p53 tumor suppressor gene in carcinogenesis and tumor progression. FASEB J..

[189] Cook T., Wang Z., Alber S., Liu K., Watkins S.C., Vodovotz Y. (2004). Nitric oxide and ionizing radiation synergistically promote apoptosis and growth inhibition of cancer by activating p53. Cancer Res..

[190] Ortega A.L., Mena S., Estrela J.M. (2010). Oxidative and nitrosative stress in the metastatic microenvironment. Cancers (Basel).

[191] Ahuja S., Sureka N., Zaheer S. (2024). Unraveling the intricacies of cancer-associated fibroblasts: a comprehensive review on metabolic reprogramming and tumor microenvironment crosstalk. APMIS.

[192] Suman I., Simic L., Canadi Juresic G., Buljevic S., Klepac D., Domitrovic R. (2024). The interplay of mitophagy, autophagy, and apoptosis in cisplatin-induced kidney injury: involvement of ERK signaling pathway. Cell Death Discov..

[193] Hanahan D. (2022). Hallmarks of cancer: new dimensions. Cancer Discov..

[194] Ma C., Hu H., Liu H., Zhong C., Wu B., Lv C. (2025). Lipotoxicity, lipid peroxidation and ferroptosis: a dilemma in cancer therapy. Cell Biol. Toxicol..

[195] Xiao M., Yang H., Xu W., Ma S., Lin H., Zhu H. (2012). Inhibition of alpha-KG-dependent histone and DNA demethylases by fumarate and succinate that are accumulated in mutations of FH and SDH tumor suppressors. Genes Dev..

[196] Ramel E., Lillo S., Daher B., Fioleau M., Daubon T., Saleh M. (2021). The metabolic control of Myeloid cells in the tumor microenvironment. Cells.

[197] Switzer C.H., Ridnour L.A., Cheng R., Heinecke J., Burke A., Glynn S. (2012). S-Nitrosation mediates multiple pathways that lead to tumor progression in Estrogen receptor-negative breast cancer. For Immunopathol Dis Therap..

[198] Olson N., van der Vliet A. (2011). Interactions between nitric oxide and hypoxia-inducible factor signaling pathways in inflammatory disease. Nitric Oxide.

